# Aberrant development of pancreatic beta cells derived from human iPSCs with *FOXA2* deficiency

**DOI:** 10.1038/s41419-021-03390-8

**Published:** 2021-01-20

**Authors:** Ahmed K. Elsayed, Ihab Younis, Gowher Ali, Khalid Hussain, Essam M. Abdelalim

**Affiliations:** 1 Diabetes Research Center, Qatar Biomedical Research Institute (QBRI), Hamad Bin Khalifa University (HBKU), Qatar Foundation (QF), PO Box 34110, Doha, Qatar; 2 Biological Sciences Program, Carnegie Mellon University, Qatar Foundation, Education City, Doha, Qatar; 3 Division of Endocrinology, Department of Pediatric Medicine, Sidra Medicine, Doha, Qatar; 4 College of Health and Life Sciences, Hamad Bin Khalifa University (HBKU), Qatar Foundation, Education City, Doha, Qatar

**Keywords:** Induced pluripotent stem cells, Diabetes

## Abstract

FOXA2 has been identified as an essential factor for pancreas development and emerging evidence supports an association between FOXA2 and diabetes. Although the role of FOXA2 during pancreatic development is well-studied in animal models, its role during human islet cell development remains unclear. Here, we generated induced pluripotent stem cells (iPSCs) from a patient with *FOXA2* haploinsufficiency (FOXA2^+/^^−^ iPSCs) followed by beta-cell differentiation to understand the role of FOXA2 during pancreatic beta-cell development. Our results showed that *FOXA2* haploinsufficiency resulted in aberrant expression of genes essential for the differentiation and proper functioning of beta cells. At pancreatic progenitor (PP2) and endocrine progenitor (EPs) stages, transcriptome analysis showed downregulation in genes associated with pancreatic development and diabetes and upregulation in genes associated with nervous system development and WNT signaling pathway. Knockout of FOXA2 in control iPSCs (FOXA2^−/−^ iPSCs) led to severe phenotypes in EPs and beta-cell stages. The expression of NGN3 and its downstream targets at EPs as well as INSUILIN and GLUCAGON at the beta-cell stage, were almost absent in the cells derived from FOXA2^−/−^ iPSCs. These findings indicate that FOXA2 is crucial for human pancreatic endocrine development and its defect may lead to diabetes based on FOXA2 dosage.

## Introduction

During human development, early endodermal tissue becomes specified toward a pancreatic fate before evagination of pancreatic buds, populated with pancreatic progenitors. All adult pancreatic cells are originated from the same progenitors expressing a group of transcription factors (TFs), including PDX1, SOX9, FOXA2, NKX6.1, HNF6, and PTF1A^1,2^. Monogenic diabetes (MD) is caused by a mutation or defect in a single gene-regulating beta-cell development and/or function^3^. Several heterozygous mutations in the TFs expressed during pancreatic development are associated with a specific form of MD, known as MODY. However, homozygous mutations of the same TFs lead to neonatal diabetes, which can be associated with pancreatic hypoplasia/agenesis in some mutations^4^. This indicates that the onset and severity of the diabetes phenotype are correlated with the dosage of the TF expression during pancreatic development.

FOXA2 is expressed in several tissues and performs distinct functions as evident in the phenotypes of mouse models^5^. *Foxa2* knockout mice die at an early embryonic stage and show developmental defects in the foregut (FG) and neural tube^6–8^. During pancreatic development, FOXA2 is expressed at early stages starting from the endoderm stage and its protein level is increased during the endocrine specification stage^1,9^, while the exocrine and ductal cells express a low level of FOXA2^9^. Mouse studies showed that Foxa2 is important for islet development and beta-cell functionality^6,10–12^ and its specific deletion in beta-cells leads to hyperinsulinemic hypoglycemic phenotype^13,14^. In human, previous reports demonstrated that patients with heterozygous *FOXA2* mutations develop hyperinsulinemia, hypoglycemia, hypopituitarism, endodermal organ defects, and craniofacial abnormalities^15,16^. Recent genomic studies found that type 2 diabetes (T2D) risk alleles are associated with FOXA2-bound enhancers in human^17^. Another recent study reported a patient with diabetes due to a heterozygous missense variant in *FOXA2*, indicating that FOXA2 defect can lead to MD^18^. FOXA2 is known to regulate the expression of multiple TFs controlling pancreatic endocrine cell fate and insulin secretion^19,20^.

Most of the information available on FOXA2 functions have been obtained from animal studies^7,10,11,13,21^, which do not fully reflect human phenotypes. Recent progress in the induced pluripotent stem cell (iPSC) technology allowed us to generate in vitro models to study human genetic diseases^22^. To our knowledge, there is only one recent study that used *FOXA2* knockout hESCs (FOXA2^−/−^ hESCs) to study FOXA2 during pancreatic progenitor differentiation^23^. To better understand the role of FOXA2 in beta cell development and diabetes progression, we established iPSCs from a patient with a heterozygous deletion in *FOXA2* (FOXA2^+/−^ iPSCs)^24^. We showed a number of key genes essential for pancreatic development were dysregulated in FOXA2^+/−^ iPSC-derived pancreatic cells and the results obtained were further validated by generating CRISPR/Cas9-mediated FOXA2 knockout (FOXA2^−/−^ iPSCs). Our results suggest that FOXA2 have a crucial role in pancreatic endocrine development in human.

## Results

### Derivation of iPSCs from a patient with FOXA2 heterozygous deletion

Recently, we reported the generation of an iPSC line from a patient with a heterozygous deletion in *FOXA2* gene (QBRIi009-A; FOXA2^+/−^ iPSC-C1)^24^ and from a healthy control (QBRIi001; Ctr1-iPSCs)^25^. In this study, we generated additional iPSC lines from another healthy control (Ctr2-iPSCs). All the established iPSC lines showed the expression of the pluripotency markers and showed the ability to differentiate into the three germ layers (Supplementary Figs. S1 and S2). Ctr-iPSC lines showed normal karyotype, while FOXA2^+/−^ iPSC lines showed a translocation between the short arm of chromosome 6 and 20, karyotype: [46 XY, t (6; 20) (p11; p11)] and a deletion [46, XY, del (20) (p11.21p11.22)] as expected^24^ and as reported in the patient sample^26^ (Supplementary Fig. S1E). Three well-characterized clones from FOXA2^+/−^ iPSCs were utilized for the subsequent experiments.

### Effect of FOXA2 haploinsufficiency on definitive endoderm (DE) formation

**Fig. 1:**
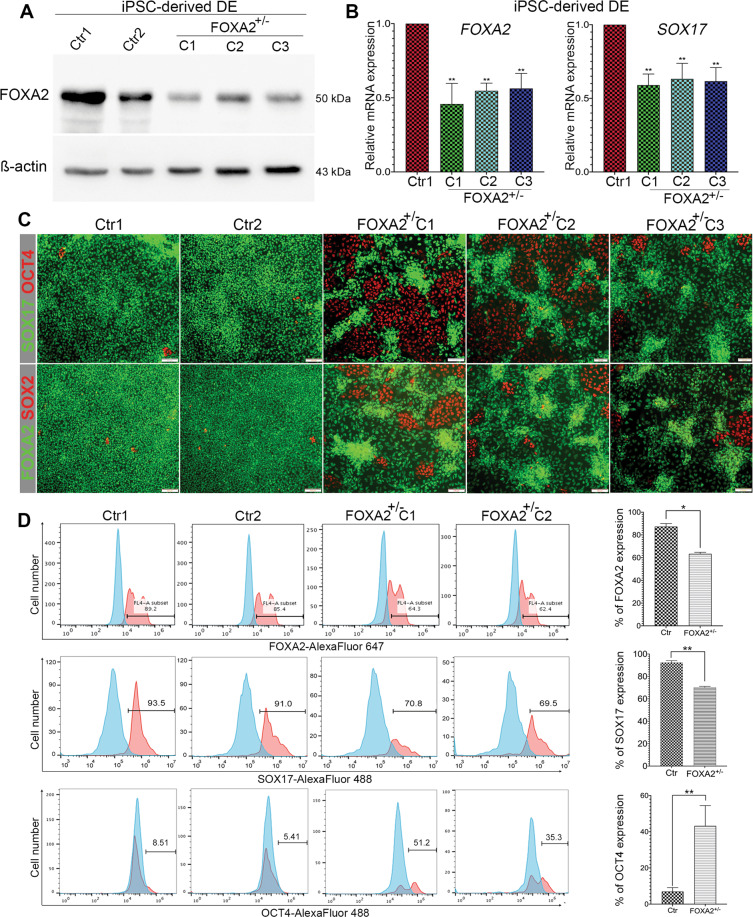
Effect of *FOXA2* haploinsufficiency on definitive endoderm (DE) formation. **A** Western blotting of FOXA2 expression in the definitive endoderm (DE) derived from Ctr-iPSCs and FOXA2^+/−^ iPSCs. **B** qPCR quantification of the expression of DE markers, *FOXA2* and *SOX17* in DE derived from Ctr-iPSCs and FOXA2^+/−^ iPSCs (*n* = *3*). **C** Representative immunofluorescent images showing the expression of SOX17 and OCT4 or FOXA2 and SOX2 in DE derived from Ctr-iPSCs and FOXA2^+/−^ iPSCs. **D** Representative flow cytometry histograms and its quantification showing the expression of FOXA2, SOX17, and OCT4 in DE derived from Ctr-iPSCs and FOXA2^+/−^ iPSCs. Data are represented as mean ± SD; *p < 0.05, **p < 0.01, ***p < 0.001.

To investigate the effect of *FOXA2* heterozygous deletion on pancreatic beta-cell development, Ctr-iPSCs and three FOXA2^+/−^ iPSC lines were differentiated into all stages of beta cells as previously reported^27–29^ (Supplementary Fig. S3A). Since FOXA2 expression starts at the definitive endoderm (DE) stage, we examined the impact of *FOXA2* haploinsufficiency on DE formation. The protein and mRNA levels of FOXA2 were significantly reduced in DE derived from the three FOXA2^+/−^ iPSC lines in comparison to those derived from Ctr-iPSC lines (Fig. 1A–D), confirming *FOXA2* haploinsufficiency. qPCR analysis showed a significant decrease in the expression of SOX17, the DE marker (Fig. 1B). Furthermore, immunostaining and flow cytometry analyses showed a marked reduction in the expression of the DE markers, SOX17 and FOXA2 and a significant increase in the expression of the pluripotency markers, OCT4 and SOX2 in the DE derived from the FOXA2^+/−^ iPSC lines compared to those derived from Ctr-iPSCs (Fig. 1C, D). OCT4^+^ cells ranged from 35 to 51% in the DE derived from FOXA2^+/−^ iPSCs compared to 5–9% in those derived from Ctr-iPSCs (Fig. 1D) indicating the retention of the pluripotency markers and a delay in DE formation as a result of *FOXA2* deficiency.

### FOXA2 deficiency leads to aberrant development of PDX1^+^/NKX6.1^+^ progenitors and endocrine progenitors

**Fig. 2:**
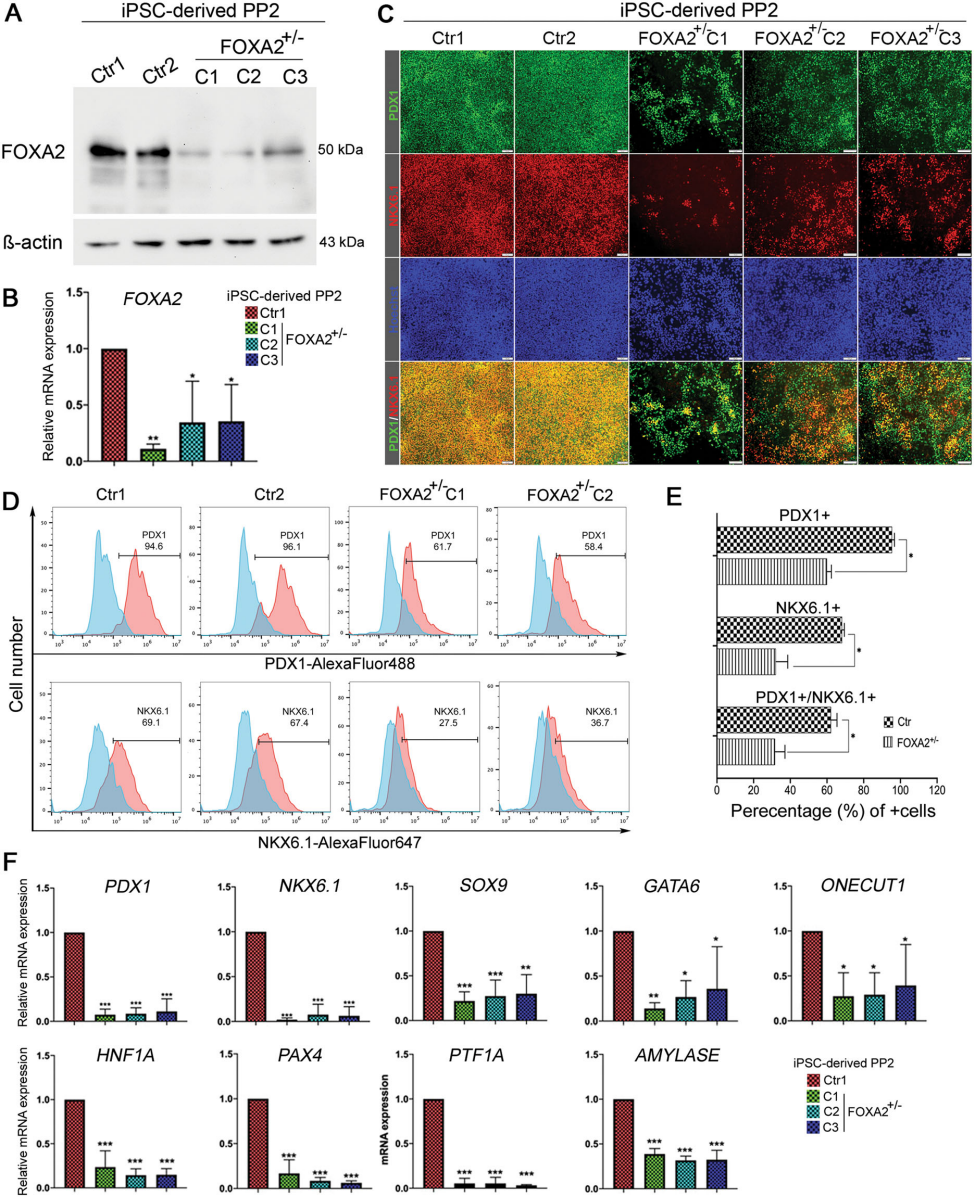
Effect of *FOXA2* haploinsufficiency on pancreatic progenitor (PP2) differentiation. Western blotting (**A**) and qPCR analysis (**B**) for FOXA2 protein and mRNA expression levels in iPSC-derived PP2. **C** Representative immunofluorescent images showing the expression of PDX1 and NKX6.1 in the PP2 derived from Ctr-iPSCs and FOXA2^+/−^ iPSCs. **D** Flow cytometry histograms showing the expression of PDX1 and NKX6.1 in the PP2 derived from Ctr-iPSCs and FOXA2^+/−^ iPSCs. **E** Quantification of the expression of PDX1 and NKX6.1 shown in **D**. **F** qPCR quantification for the expression of key PP2 markers, *PDX1*, *NKX6.1*, *SOX9*, *HNF1A*, *GATA6*, *PAX4*, *ONECUT1 (HNF6)*, *PTF1A*, and *AMYLASE* in the generated PP2. Data are represented as mean ± SD; *p < 0.05, **p < 0.01, ***p < 0.001.

We next sought to assess the impact of *FOXA2* deficiency on the differentiation of pancreatic progenitors (PP2) and endocrine progenitors (EPs). FOXA2 protein and mRNA levels were markedly reduced in PP2 derived from FOXA2^+/−^ iPSCs (Fig. 2A, B), confirming the haploinsufficiency. We noticed that the efficiency of PP2 was negatively impacted as a result of FOXA2 reduction. The immunostaining and flow cytometry analyses showed a significant decrease in the expression of the key pancreatic progenitor TFs, PDX1 and NKX6.1 (above 35% reduction in PDX1 and NKX6.1) (Fig. 2C–E), further confirmed at mRNA levels using qPCR (Fig. 2F). The co-expression of PDX1 and NKX6.1 in the PP2 is known to be the origin of pancreatic beta cells^27,28^. Furthermore, we investigated the mRNA expression levels of other TFs, expressed at the PP2 stage and are important for beta-cell development, including *SOX9*, *GATA6*, *ONECUT1 (HNF6)*, *HNF1A*, and *PAX4 as well as* the exocrine precursor markers, *PTF1A* and *AMYLASE*. Interestingly, all these pancreatic markers were significantly downregulated in PP2 derived from FOXA2^+/−^ iPSC lines compared to those derived from Ctr-iPSCs (Fig. 2F), indicating the importance of FOXA2 in PP2.

**Fig. 3:**
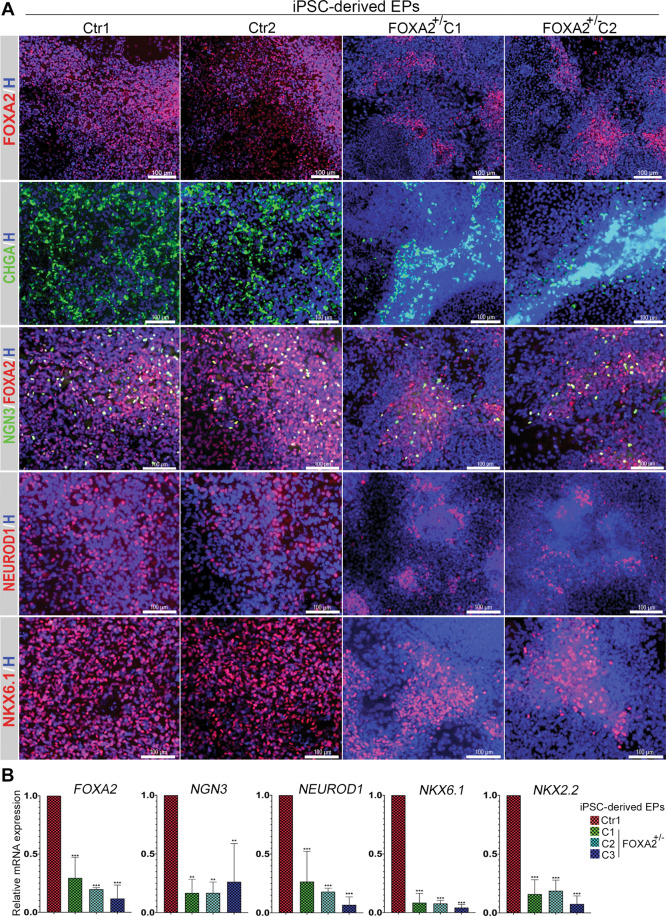
Effect of *FOXA2* haploinsufficiency on pancreatic endocrine progenitors (EPs) differentiation. **A** Representative immunofluorescent images showing the expression of FOXA2, NGN3, CHGA, NEUROD1, and NKX6.1 in endocrine progenitors (EPs) derived from Ctr-iPSCs and FOXA2^+/−^ iPSCs. **B** Real-time PCR analysis for the expression of the key EPs markers, *FOXA2*, *NGN3*, *CHGA*, *NEUROD1*, *NKX6.1*, and *NKX2.2* in the EPs derived from Ctr-iPSCs and FOXA2^+/−^ iPSCs. Data are represented as mean ± SD; *p < 0.05, **p < 0.01, ***p < 0.001.

To investigate the effect of *FOXA2* deficiency on EPs, the PP2 were further differentiated into EPs. The downregulation of FOXA2 was confirmed at the end of stage 5 (Fig. 3A, B). The immunostaining showed a decrease in the expression of the endocrine marker, chromogranin A (CHGA), as a result of FOXA2 deficiency (Fig. 3A). Also, the expression levels of NEUROGENIN 3 (NGN3), a crucial TF for endocrine specification, and its downstream targets, NEUROD1 and NKX2.2, as well as NKX6.1, were markedly reduced in the EPs derived from FOXA2^+/−^ iPSCs compared to those generated from Ctr-iPSCs as indicated by immunostaining and qPCR (Fig. 3A, B). Furthermore, we examined the effect of *FOXA2* deficiency on FOXA1 expression at PP2 and EPs. We found that *FOXA2* deficiency resulted in a significant reduction in the *FOXA1* mRNA levels (Supplementary Fig. S3B), suggesting a lack of a compensatory mechanism.

### Transcriptomic alterations in PP2 and EPs derived from FOXA2^+/−^ iPSCs

**Fig. 4:**
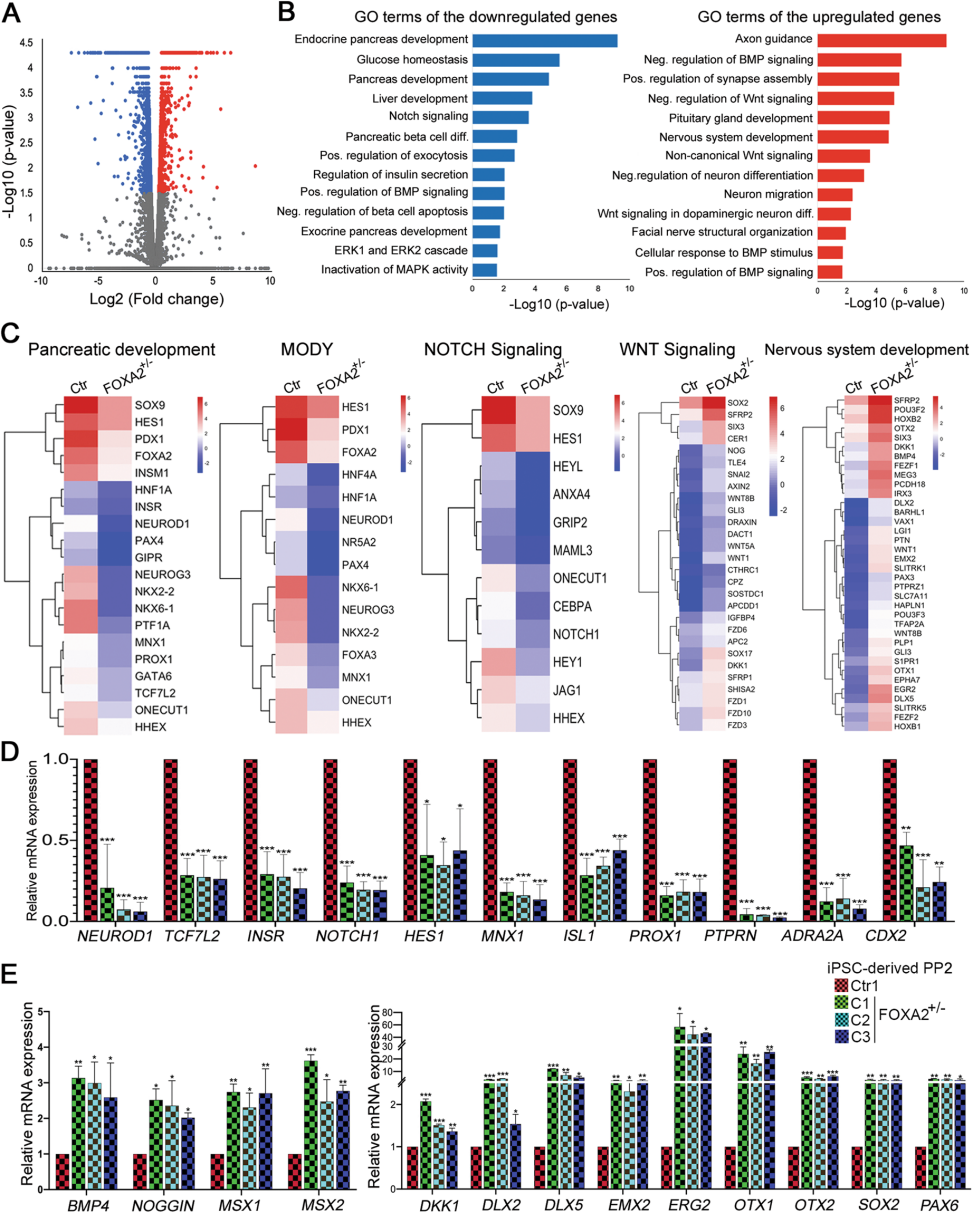
Transcriptomic alterations in pancreatic progenitors (PP2) derived from FOXA2^+/−^ iPSCs. **A** Volcano plot plotting the log2 fold change (FC) and the adjusted *p*-value for all the transcripts. **B** Pathway enrichment analysis on the upregulated and downregulated differentially expressed genes (DEGs) in FOXA2^+/−^ iPSC-derived pancreatic progenitors (PP2) using the DAVID function annotation tool. The enriched pathways were plotted against −log10 (*p*-value). **C** Heatmaps of pathway-associated DEGs in PP2 derived from FOXA2^+/−^ iPSCs compared to those derived from Ctr-iPSCs. The downregulated genes were associated with pancreatic development, maturity-onset diabetes of the young (MODY), and NOTCH signaling pathways, while the upregulated genes were associated with the WNT signaling pathway and nervous system development. The relative value for each gene is depicted by color intensity, with red indicating upregulated and blue indicating downregulated genes. RNA-Seq results were validated using qPCR for the downregulated (**D**) and the upregulated genes (**E**). Data are represented as mean ± SD; *p < 0.05, **p < 0.01, ***p < 0.001.

To gain further insight into the gene regulatory network influenced by *FOXA2* leading to progenitor development defects, we performed whole-transcriptome profiling using RNA-Seq analysis on PP2 and EPs. Our transcriptome analysis identified 610 downregulated (Log2 FC < −1.0, *p* < 0.05) and 427 upregulated (Log2 FC > 1.0, *p* < 0.05) differentially expressed genes (DEGs) in PP2 derived from FOXA2^+/−^ iPSCs compared to those generated from Ctr-iPSCs (Fig. 4A). To investigate biological functions and pathways enriched in FOXA2-deficient PP2, GO and KEGG pathway enrichment analyses were performed. The enriched pathways of the downregulated DEGs were mainly associated with pancreatic development, liver development, glucose homeostasis, insulin secretion, MODY, T2D, and NOTCH signaling (Fig. 4B, C and Supplementary Table S4). However, the upregulated DEGs led to several enriched pathways, such as axon guidance, nervous system development, and WNT and BMP signaling (Fig. 4B, C and Supplementary Fig. S3C, Supplementary Table S5). Consistent with the qPCR results in Fig. 2F, the main PP2-specific TFs were significantly downregulated, such as *PDX1*, *NKX6.1*, *FOXA2*, *SOX9*, *GATA6*, *PAX4*, *HNF6*, and *PTF1A* (Figs. 2F, 4C and Supplementary Table S4). Furthermore, several genes involved in the endocrine specification and diabetes development were significantly downregulated, such as *HES1*, *INSM1*, *HNF1A*, *NEUROD1*, *NGN3*, *NKX2.2*, *GIPR*, *MNX1*, *PROX1, TCF7L2*, and *HHEX* (Fig. 4C and Supplementary Table S4). Consistent with pancreatic developmental defects, we noticed a significant reduction in the genes involved in the NOTCH signaling pathway, such as *SOX9*, *NOTCH1*, *HES1*, and *HEY1*, which have a key role in PP2 differentiation (Fig. 4C and Supplementary Table S4).

To validate the RNA-Seq data, we examined the mRNA expression levels of selected DEGs. qPCR showed a significant reduction in *NEUROD1, TCF7L2, INSR, NOTCH1*, *HES1, MNX1, ISL1*, *PROX1*, *PTPRN*, *ADRA2A*, and *CDX2* (Fig. 4D). Furthermore, the results showed a significant upregulation in *BMP4*, *NOGGIN*, *MSX1, MSX2*, *DKK1*, *DLX2*, *DLX5*, *EMX2*, *ERG2, OTX1*, *OTX2*, *SOX2*, and *PAX6* (Fig. 4E). These results are consistent with the RNA-Seq data.

**Fig. 5:**
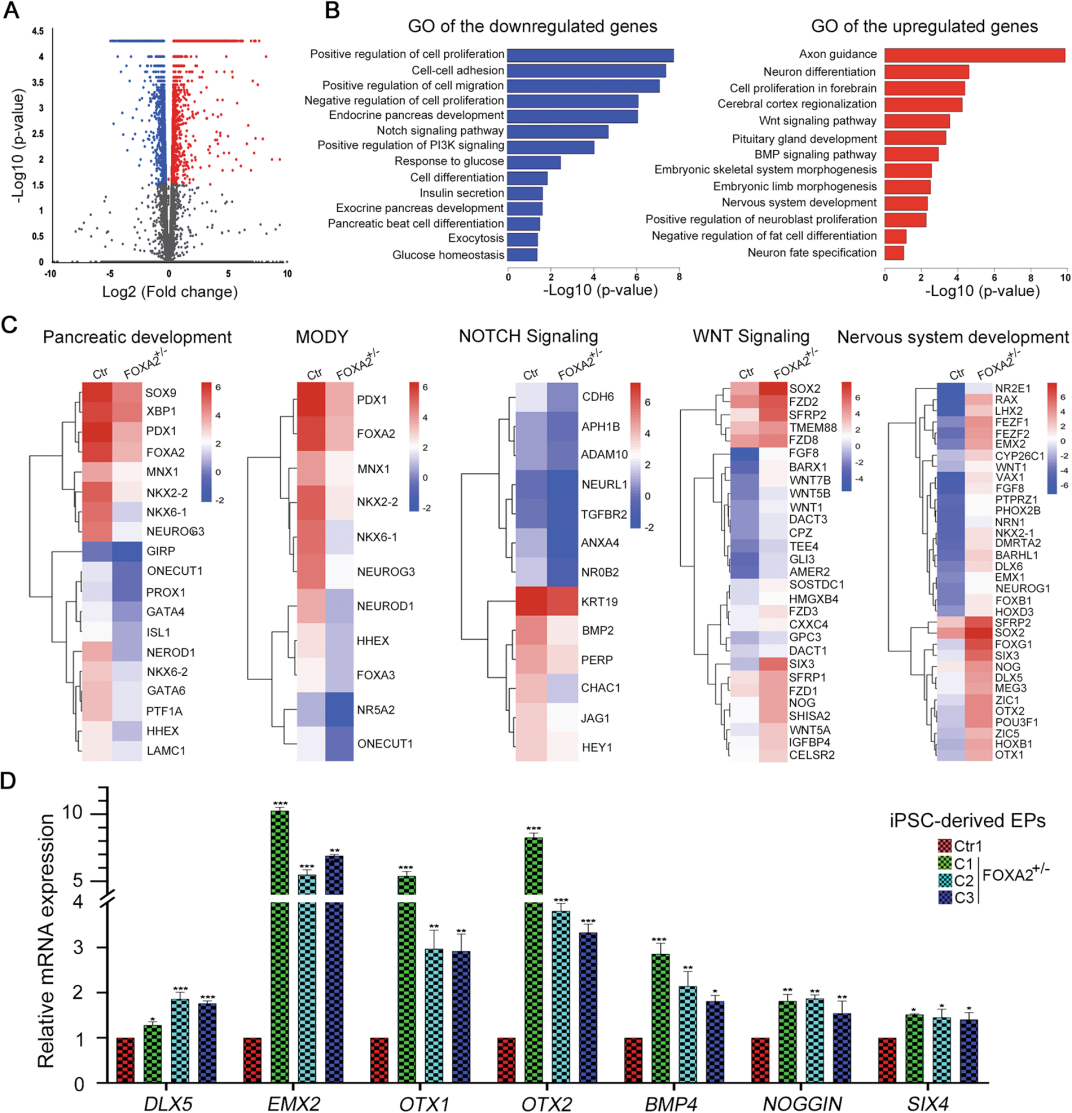
Transcriptomic changes in pancreatic endocrine progenitors (EPs) derived from FOXA2^+/−^ iPSCs. **A** Volcano plot plotting the log2 fold change (FC) and the adjusted *p*-value for all the transcripts. **B** Pathway enrichment analysis on upregulated and downregulated differentially expressed genes (DEGs) in FOXA2^+/−^ iPSC-derived EPs using DAVID function annotation tool. The enriched pathways were plotted against −log10 (*p*-value). **C** Heatmaps of pathway-associated DEGs in FOXA2^+/−^ iPSC-derived EPs compared to those derived from Ctr-iPSCs. The downregulated genes were associated with pancreatic development, maturity-onset diabetes of the young (MODY), and NOTCH signaling pathways, while the upregulated genes were associated with the WNT signaling pathway and nervous system development. The relative value for each gene is depicted by color intensity, with red indicating upregulated and blue indicating downregulated genes. **D** RNA-Seq results were validated using qPCR for the upregulated genes associated with WNT signaling and nervous system development. Data are represented as mean ± SD; *p < 0.05, **p < 0.01, ***p < 0.001.

Our RNA-Seq analysis on EPs identified 643 downregulated (Log2 FC < −1.0, *p* < 0.05) and 564 upregulated (Log2 FC > 1.0, *p* < 0.05) DEGs in EPs derived from FOXA2^+/−^ iPSCs compared to those generated from Ctr-iPSCs (Fig. 5A). The enriched pathways of the downregulated and upregulated DEGs were similar to those observed in PP2 (Fig. 5B). The downregulated DEGs were mainly associated with endocrine pancreas development, NOTCH signaling pathway, MODY, and insulin secretion and glucose homeostasis (Fig. 5B, C and Supplementary Table S6). However, the upregulated DEGs were mainly associated with neurogenesis and nervous system development, WNT pathway, and BMP signaling pathway (Fig. 5B, C and Supplementary Fig. S3D, Supplementary Table S7). In addition to the downregulated pancreatic endocrine TFs shown in Fig. 3B, we further validated selected upregulated DEGs using qPCR. Consistent with RNA-Seq data, the mRNA levels of *DLX5*, *EMX2*, *OTX1*, *OTX2*, *BMP4*, *NOGGIN*, and *SIX4* were significantly upregulated in the EPs derived from FOXA2^+/−^ iPSCs compared to those derived from Ctr-iPSCs (Fig. 5D).

### Effect of FOXA2 haploinsufficiency on beta-cell differentiation

**Fig. 6:**
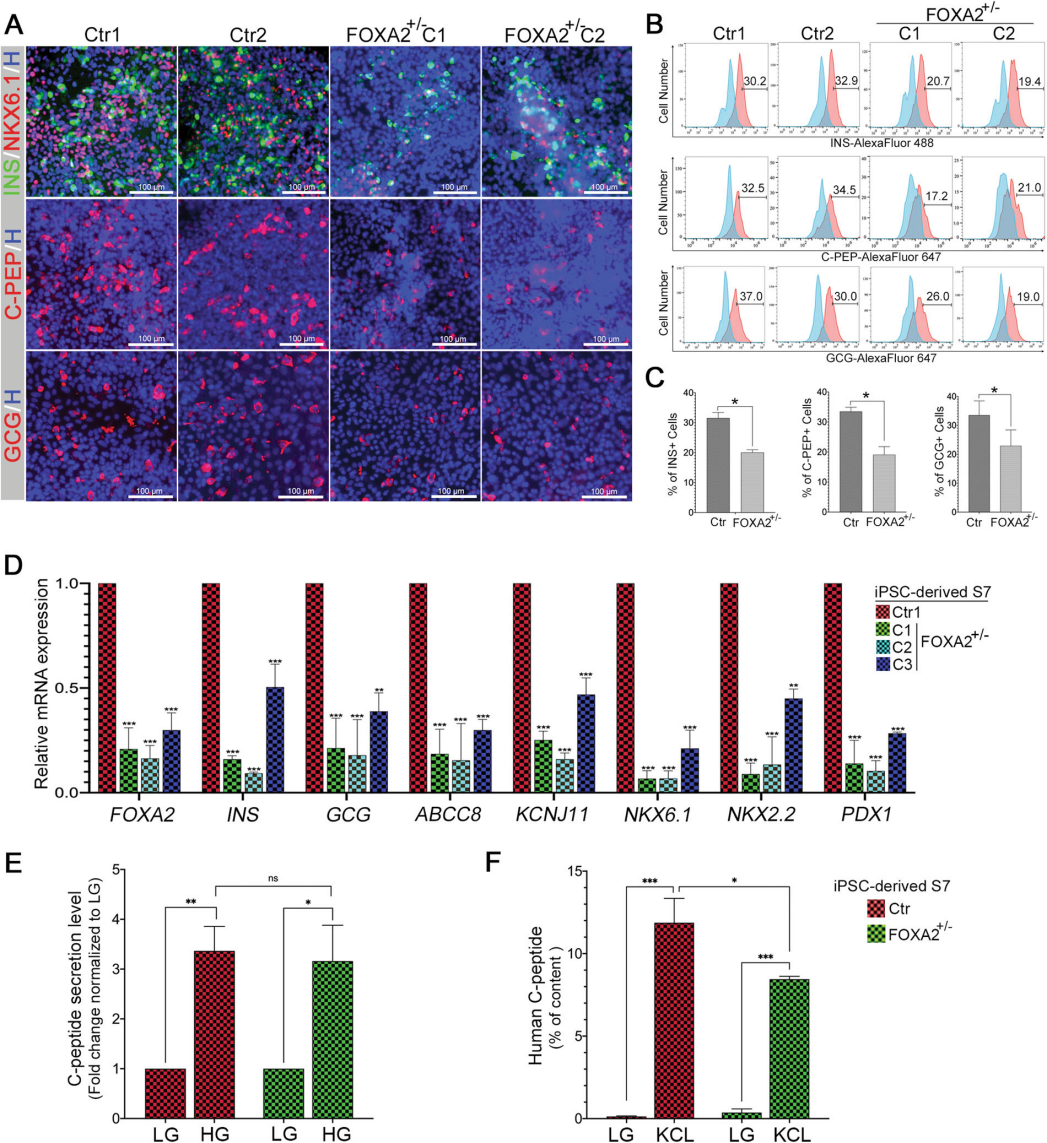
Effect of *FOXA2* haploinsufficiency on iPSC-derived beta cells. Representative immunofluorescent images (**A**) and flow cytometry histograms (**B**) showing the expression of INSULIN (INS), NKX6.1, C-PEPTIDE (C-PEP), and GLUCAGON (GCG) in stage 7 derived from Ctr-iPSCs and FOXA2^+/−^ iPSCs. **C** Flow cytometry quantification for the data shown in **B**. **D** qPCR quantification for the expression of the pancreatic markers, *FOXA2, INS, GCG, ABCC8, KCNJ11, NKX6.1, NKX2.2*, and *PDX1* in beta cells derived from Ctr-iPSCs and FOXA2^+/−^ iPSCs. GSIS assay: fold change of C-PEPTIDE secretion in iPSC-derived beta cells challenged with low (LG; 2.8 mM) and high glucose (HG; 20 mM) (**E**) or stimulated with or without 30 mM KCl in the presence of LG (**F**). Data are represented as mean ± SD; *p < 0.05, **p < 0.01, ***p < 0.001.

In order to investigate the influence of *FOXA2* haploinsufficiency on beta cells, the EPs were further differentiated into beta cells. Ctr-iPSCs and FOXA2^+/−^ iPSCs were able to generate INSULIN (INS)^+^ cells. However, the expression levels of INS, NKX6.1, C-PEP, and GLUCAGON (GCG) were downregulated as examined by immunostaining and flow cytometry in stage 7 derived from FOXA2^+/−^ iPSCs compared to those derived from Ctr-iPSCs (Fig. 6A–C). The expression of NKX6.1 in INS^+^ cells, one of the hallmarks of the functionality of beta cells, was also reduced. Furthermore, the mRNA levels of *ABCC8*, *KCNJ11*, *NKX2.2*, and *PDX1*, which are known to be regulated by *FOXA2*^19,20^ were significantly downregulated due to *FOXA2* haploinsufficiency (Fig. 6D). These results indicate that the number of beta and alpha cells are reduced as a result of *FOXA2* haploinsufficiency.

To test the functionality of the generated beta cells, we performed a GSIS assay. The ability of iPSC-derived beta-cell aggregates to secrete C-PEP in response to low (2.8 mM) and high (20 mM) glucose and KCl stimulation was measured. Beta cells derived from FOXA2^+/−^ iPSCs and Ctr-iPSCs showed a significant increase in the secreted C-PEP in response to high glucose (FC > 3.0) and KCl stimulation compared to low glucose levels (Fig. 6E, F). We did not notice a significant difference in glucose responsiveness between the beta cells derived from FOXA2^+/−^ iPSCs and those derived from Ctr-iPSCs (Fig. 6E). However, KCl stimulation showed a significant reduction in the C-PEP content in beta cells derived from FOXA2^+/−^ iPSCs compared with those derived from Ctr-iPSCs (Fig. 6E). These results indicate that *FOXA2*-deficient beta cells are functional, but the total content of insulin is reduced as a result of a lower number of beta cells due to *FOXA2* haploinsufficiency.

### FOXA2 knockout in iPSCs impairs endocrine pancreatic development

**Fig. 7:**
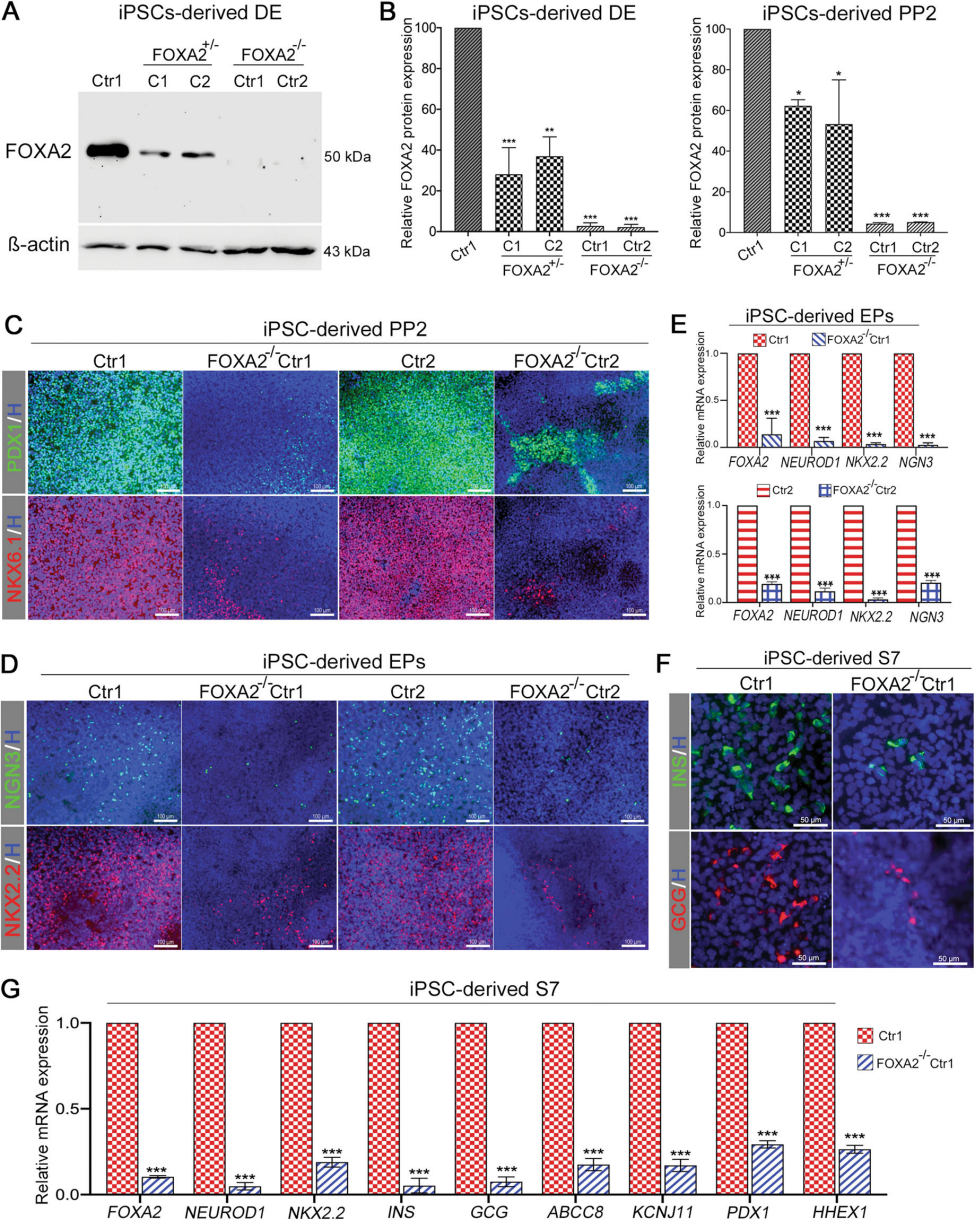
Effect of FOXA2 knockout on iPSC-derived beta cells. **A** Western blotting of FOXA2 expression in the definitive endoderm (DE) derived from Ctr-iPSCs, FOXA2^+/−^ iPSCs, and FOXA2^−/−^ iPSCs. **B** Quantification of FOXA2 protein levels in DE and PP2 derived from Ctr-iPSCs, FOXA2^+/−^ iPSCs, and FOXA2^−/−^ iPSCs. Note the complete absence of FOXA2 expression in the DE derived from FOXA2^−/−^ iPSCs. Representative immunofluorescence images showing the expression of PDX1 and NKX6.1 in PP2 (**C**) and NGN3 and NKX2.2 in EPs (**D**) derived from WT controls and FOXA2^−/−^ iPSCs. **E** Real-time PCR analysis for the expression of *FOXA2*, *NEUROD1*, *NKX2.2*, and *NGN3* in EPs derived from FOXA2^−/−^ iPSCs and their WT controls. **F** Representative immunofluorescence images showing the expression of INS and GCG in stage 7 derived from FOXA2^−/−^ iPSCs and Ctr1-iPSCs. **G** qPCR quantification for the expression of *FOXA2*, *NEUROD1*, *NKX2.2*, *INS*, *GCG*, *ABCC8*, *KCNJ11*, *PDX1*, and *HHEX* in stage 7 derived from FOXA2^−/−^ iPSC line and its WT control, Ctr1-iPSCs. The data are presented as mean ± SD. *p < 0.05, **p < 0.01, ***p < 0.001.

To confirm the results obtained from the *FOXA2*^+/−^ iPSCs and to exclude the possibility of cell line variations, we used CRISPR/Cas9 to KO the *FOXA2* gene in the iPSCs (FOXA2^−/−^ iPSCs) (Supplementary Fig. S4A–D). We generated FOXA2^−/−^ iPSCs from Ctr1-iPSCs and Ctr2-iPSCs used in the current study and compared them with isogenic FOXA2^+/+^ wild type (WT) controls. Characterization of both FOXA2^−/−^ iPSC lines showed that they were pluripotent and displayed normal karyotype (Supplementary Fig. S4E). Next, FOXA2^−/−^ iPSC lines and their WT controls were differentiated into beta cells. Western blotting showed that the FOXA2 protein was completely lost in the DE and PP2 derived from FOXA2^−/−^ iPSC lines (Fig. 7A, B). Consistent with the results obtained from *FOXA2* haploinsufficiency, the expression of the key TFs, PDX1 and NKX61 were markedly reduced in PP2 derived from FOXA2^−/−^ iPSC lines compared to WT iPSCs as examined by immunostaining (Fig. 7C). At the EPs stage, the immunostaining analysis showed that the depletion of FOXA2 resulted in the generation of a very low number of NGN3^+^/NKX2.2^+^ cells in comparison to WT controls (Fig. 7D). Furthermore, qPCR results showed a highly significant reduction in the expression of the main endocrine TFs, *NGN3*, *NEUROD1*, and *NKX2.2* in the EPs derived from FOXA2^−/−^ iPSC lines (Fig. 7E). Interestingly, immunostaining showed that only a small number of INS^+^ and GCG^+^ cells were detected in FOXA2^−/−^ iPSC-derived beta cells compared to WT (Fig. 7F). Consistent with these findings, qPCR showed significant downregulation in the endocrine and beta-cell genes, *NEUROD1*, *NKX2.2*, *INS*, *GCG*, *ABCC8*, *KCJ11*, *PDX1*, and *HHEX1* (Fig. 7G). Our results showed that the FOXA2^−/−^ phenotypes were more severe than those obtained from FOXA2^+/−^ iPSCs indicating that FOXA2 is required for endocrine pancreatic development.

### Overexpression of FOXA2 in pancreatic progenitors reverses the phenotype associated with *FOXA2* deficiency

**Fig. 8:**
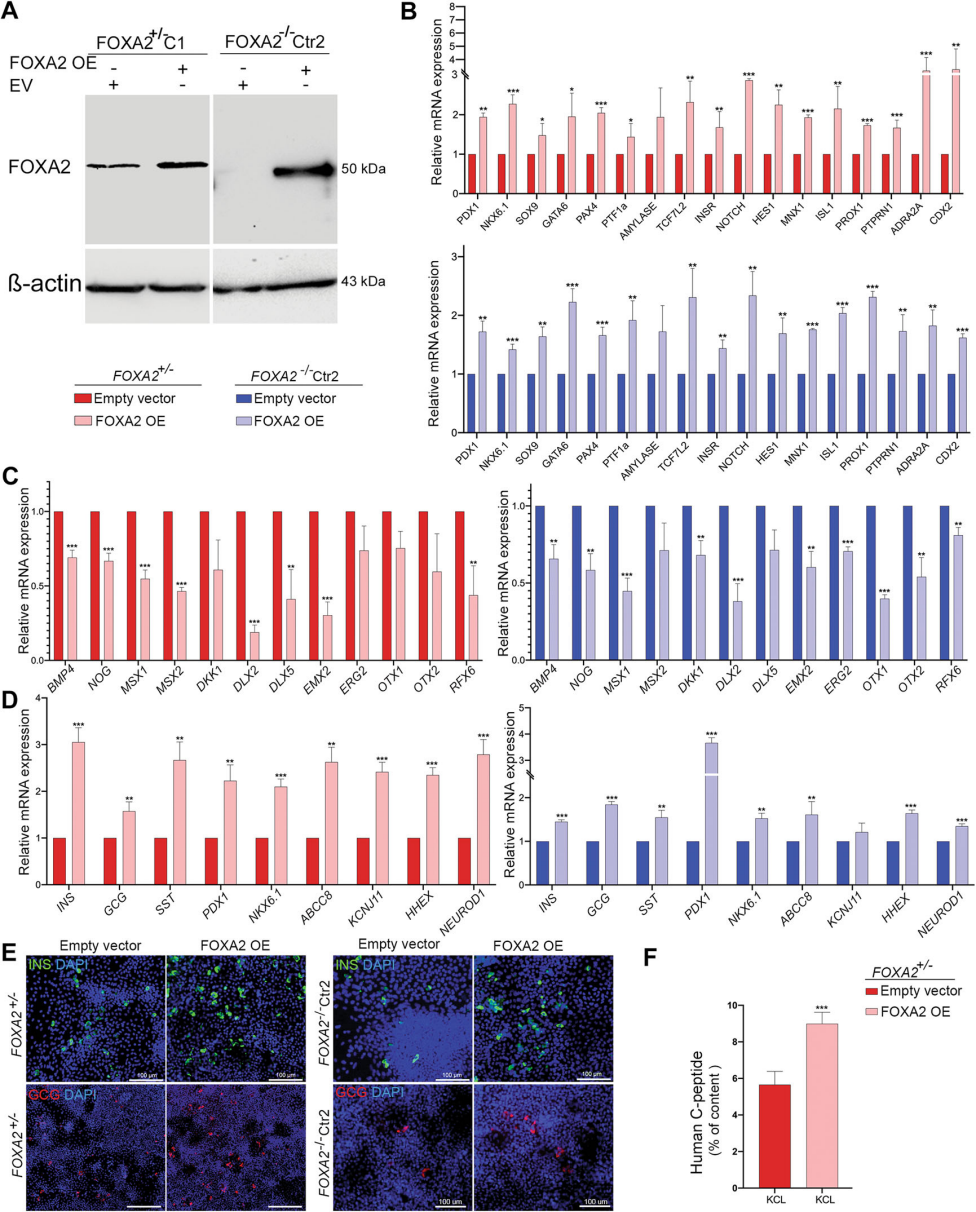
FOXA2 overexpression in PP2 reverse the phenotype of *FOXA2* deficiency. **A** Representative western blotting showing the overexpression of FOXA2 in the PP2 derived from FOXA2^+/−^ iPSCs, and FOXA2^−/−^ iPSCs, 48 h after transfection with FOXA2 plasmid or empty vector (EV). **B** Real-time PCR analysis for the expression of *PDX1*, *NKX6.1*, *SOX9*, *GATA6*, *PAX4*, *PTF1A*, *AMYLASE*, *TCF7L2*, *INSR*, *NOTCH*, *HES1*, *MNX1, ISL1, PROX1, PTPRN1, ADRA2A*, and *CDX2* in PP2 derived from FOXA2^+/−^ iPSCs and FOXA2^−/−^ iPSCs 48 h after transfection. **C** Real-time PCR analysis for the expression of *BMP4*, *NOG*, *MSX1*, *MSX2*, *DKK1*, *DLX2*, *DLX5*, *EMX2*, *ERG2*, *OTX1*, *OTX2*, and *RFX6* in PP2 derived from FOXA2^+/−^ iPSCs and FOXA2^−/−^ iPSCs 48 h after transfection. **D** Real-time PCR analysis for the expression of *INS*, *GCG*, *SST*, *PDX1*, *NKX6.1*, *ABCC8*, *KCNJ11*, *HHEX*, and *NEUROD1* in stage 7 derived from FOXA2^+/−^ iPSCs and FOXA2^−/−^ iPSCs transfected with FOXA2 plasmid or EV at stage 4. **E** Representative immunofluorescence images showing the expression of INS and GCG in stage 7 derived from FOXA2^+/−^ iPSCs and FOXA2^−/−^ iPSCs transfected with FOXA2 plasmid or EV at stage 4. **F** C-PEPTIDE (C-PEP) secretion (% of content) after stimulation with or without 30 mM KCl in the presence of low glucose (LG) in FOXA2^+/−^ iPSC-derived beta cells transfected with FOXA2 plasmid or EV at stage 4. The data are presented as mean ± SD. *p < 0.05, **p < 0.01, ***p < 0.001.

In order to examine whether the phenotype observed due to *FOXA2* deficiency can be reversed by re-introducing FOXA2 expression, we overexpressed FOXA2 at day 2 of stage 4 of differentiation (PP2 stage) derived from *FOXA2*^+/−^ iPSCs and *FOXA2*^−/−^ iPSCs (Fig. 8A). qPCR analysis at the end of stage 4 showed that FOXA2 OE resulted in a significant increase in the expression of the genes that were downregulated in *FOXA2-*deficient PP2 cells (Fig. 8B). Furthermore, FOXA2 OE led to a significant downregulation in the majority of the genes that were upregulated in *FOXA2-*deficient PP2 cells (Fig. 8C).

In order to assess the effect of the transient FOXA2 OE at the PP2 stage on iPSC differentiation towards pancreatic beta cells, we evaluated the key beta-cell markers and GSIS in stage 7 cells derived from both *FOXA2*^+/−^ iPSCs and *FOXA2*^−/−^ iPSCs after FOXA2 overexpression. At stage 7, we found that FOXA2 OE at PP2 resulted in a significant increase in the expression of the key pancreatic endocrine genes, including *INS*, *GCG*, *SST*, *PDX1*, *NKX6.1*, *ABCC8*, *KCNJ11*, *HHEX*, and *NEUROD1* in comparison to those transfected with the empty vector (Fig. 8D). Consistent with these findings, the immunostaining results showed an increase in the number of INS^+^ and GCG^+^ cells derived from *FOXA2*^+/−^ iPSCs and *FOXA2*^−/−^ iPSCs after FOXA2 OE (Fig. 8E). Since the total content of insulin was reduced in beta cells derived from FOXA2^+/−^ iPSCs (Fig. 6E), we examined the effect of FOXA2 OE on the C-PEP content. KCl stimulation showed a significant increase in the C-PEP content in beta cells derived from FOXA2^+/−^ iPSCs overexpressed with FOXA2 compared with those transfected with the empty vector (Fig. 8E). These results indicate that FOXA2 OE can reverse the phenotype observed in the *FOXA2-*deficient pancreatic cells.

### Effect of PDX1 overexpression on FOXA2-deficient pancreatic progenitors

Previous studies reported that FOXA2 controls the *PDX1* gene during pancreatic beta-cell development in rodents and humans^10,12,30^. Therefore, we thought to examine the effect of PDX1 overexpression (PDX1 OE) at the PP2 stage on reversing the phenotype associated with *FOXA2* deficiency (Supplementary Fig. S5A, B). As expected, PDX1 OE led to an increase in the mRNA expression levels of several key PP2 genes that were downregulated in *FOXA2*-deficient PP2 derived from FOXA2^+/−^ iPSCs and FOXA2^−/−^ iPSCs, such as *NKX6.1*, *GATA6*, *PAX4*, *PTF1A*, *AMYLASE*, *TCF7L2*, *INSR*, *HES1*, *MNX1*, *PROX1*, *PTPRN1*, and *ADRA2A* (Supplementary Fig. S5C, D). Furthermore, PDX1 OE led to a downregulation in the expression of some genes that were upregulated in the *FOXA2*-deficient PP2 derived from FOXA2^+/−^ iPSCs and FOXA2^−/−^ iPSCs, such as *ERG2*, *OTX1*, and *OTX2* (Supplementary Fig. S5C, D). On the other hand, some genes were not significantly changed as a result of PDX1 OE, such as *SOX9*, *ONECUT1*, *NOG*, *DKK1*, *DLX2*, *DLX5*, and *EMX2*. Interestingly, PDX1 OE led to an upregulation of BMP4 and ERG2 (Supplementary Fig. S5C, D).

### Inhibition of BMP and WNT signaling pathways in FOXA2-deficient pancreatic progenitor cells

Since FOXA2 deficiency led to a significant upregulation in the WNT and BMP signaling pathways, we thought to analyze whether inhibiting the BMP and WNT pathways are able to counteract the deleterious effects in the generation of PP2. We treated the cells with the WNT inhibitor, XAV939, for 4 days during stage 4 of differentiation (Supplementary Fig. S6A). We noticed that WNT inhibition led to a significant downregulation in the genes that were upregulated in the PP2 derived from FOXA2^+/−^ iPSCs and FOXA2^−/−^ iPSCs (Supplementary Fig. S6B). Also, WNT inhibition led to a significant increase in the expression of some genes that were downregulated in the PP2 derived from *FOXA2*^+/−^ iPSCs and FOXA2^−/−^ iPSCs, particularly *NEUROD1*, *PAX4*, *TCF7L2*, *NOTCH*, *HES1*, *MNX1*, *PROX1*, and *PTPRN1* (Supplementary Fig. S6C). This indicates that inhibition of the WNT pathway, at least, partially reversed the phenotype noticed in the *FOXA2*-deficient PP2 stage (Supplementary Fig. S6A–C).

In the current protocol, we used BMP inhibitor, NOGGIN, during stages 2, 3, and 4 of differentiation. Therefore, to test the effect of BMP inhibition dose on the PP2 phenotype, we modified the protocol by treating stage 4 cells derived from FOXA2^+/−^ iPSCs and FOXA2^−/−^ iPSCs with two different concentrations of NOGGIN, 100 and 50 ng/mL (Supplementary Fig. S6D). The high concentration of NOGGIN (100 ng/mL) resulted in a significant reduction in the BMP pathway and other genes that were upregulated in the FOXA2-deficient PP2 in comparison to those treated with 50 ng/mL (Supplementary Fig. S6E). Although the high concentration of NOGGIN increased the expression of PP2 markers, these increases was not significant for most of the genes examined and showed some variations between PP2 derived from FOXA2^+/−^ iPSCs and those derived from FOXA2^−/−^ iPSCs (Supplementary Fig. S6F).

## Discussion

The role of FOXA2 in pancreatic development has been studied in mouse models; however, the mechanism by which FOXA2 defects contribute to the pathogenesis of human diseases is still not well-known due to the lack of the appropriate model that can mimic human phenotypes. In the current study, we established a human iPSC model to gain insight into the role of FOXA2 during human pancreatic islet development and investigated the effect of *FOXA2* haploinsufficiency on pancreatic beta-cell differentiation in vitro. Our results showed that FOXA2 is critical for the pancreatic endocrine specification. Taken together with the recent findings from other groups^18,23^, we suggest that FOXA2 defects may lead to monogenic diabetes with other abnormalities.

In the current study, *FOXA2* haploinsufficiency led to a significant reduction in the expression of the key DE-specific TFs and retained the pluripotency markers in the iPSC-derived DE. This result disagrees with the recent findings obtained from FOXA2^−/−^ hESCs, which showed that the DE and FG differentiation have not been affected by a complete loss of the *FOXA2* gene^23^. However, our results are in agreement with a recent study that demonstrated that FOXA2 is a master regulator of endoderm formation and its absence in ESCs impairs the endoderm differentiation with improper activation of DE genes and sustained pluripotency markers^31^. Taken together, these data suggest a crucial role for FOXA2 during DE formation in human.

Our results obtained from the PP2 and EPs stages showed that *FOXA2* haploinsufficiency led to a significant reduction in the expression of the monogenic diabetes-associated genes, such as *PDX1, HNF4A, HNF1A, NEUROD1, NGN3, PAX4, ABCC8, KCNJ11, GATA6*, and *MNX1*. In addition, several key pancreatic development-specific TFs were significantly downregulated in PP2 and EPs derived from FOXA2^+/−^ iPSCs, such as *NKX6.1, NKX6.2, SOX9, NKX2.2, ISL1, and GATA6*, among others. Consistent with our findings, in mouse, Foxa2 has been found to control the expression of the majority of those TFs, including *Pdx1, Nkx6.1, Nkx2.2, Hnf1a, Hnf4a, Kcnj11*, and *Abcc8*^19,32^. Furthermore, a recent study reported a significant reduction in the number of PDX1^+^ pancreatic progenitors derived from FOXA2^−/−^ hESCs^23^. Given the important role of PDX1 in regulating the expression of several genes that are critical for pancreatic development and endocrine specification^33,34^ and the fact that PDX1 is the downstream target of FOXA2^30^, our results suggest that the effect of FOXA2 on PDX1 at the PP2 stage is one of the key mechanisms by which FOXA2 control pancreatic endocrine development. In addition, at the PP2 stage, *FOXA2* haploinsufficiency resulted in a significant reduction in the NOTCH signaling pathway, which is important for early pancreatic development by enhancing PP2 expansion. Notch1 is expressed in the mouse pancreatic progenitors and activates the downstream target genes, Hes1 and Hey1^35,36^. Interestingly, we observed that *NOTCH1*, *HES1*, and *HEY1* were significantly downregulated at the PP2 stage as a result of FOXA2 deficiency. Although early studies showed that Notch inactivation enhances Ngn3 and activates premature endocrine pancreatic differentiation^37^, recent studies reported that Notch is required for pancreatic endocrine specification by activating the expression of Nkx6.1 and Ngn3 activator, Sox9^38,39^. Consistent with these findings, in addition to NOTCH pathway inhibition, we noticed downregulation of endocrine-specific TFs, such as *SOX9, NKX6.1, NGN3, NKX2.2, NEUROD1*, and *PAX4* as a result of FOXA2 deficiency.

Furthermore, our results demonstrated that *FOXA2* haploinsufficiency resulted in significant upregulation in the genes related to WNT and BMP signaling pathways at PP2 and EPs. Considering that WNT inhibition is crucial for endocrine development^40^ and BMP signaling inhibition is required for PDX1 induction in pancreatic progenitors^41^, we anticipate that increased both pathways are associated with the suppression of the pancreatic differentiation due to *FOXA2* haploinsufficiency. Confirming this concept, we demonstrated that inhibition of WNT or BMP pathways during the PP2 stage partially rescued the phenotype associated with *FOXA2* deficiency. The complete absence of FOXA2 had a more marked effect on beta-cell differentiation, particularly after the PP2 stage. FOXA2^−/−^ iPSCs showed severe impairment in generating INS^+^ and GCG^+^ cells. These findings indicate that the severity of the phenotypes is associated with the dosage of FOXA2 expression. Transient FOXA2 OE in PP2 derived from FOXA2^+/−^ iPSCs and FOXA2^−/−^ iPSCs significantly upregulated the key pancreatic development-specific genes at PP2 and beta-cell stages and significantly downregulated the expression of WNT and BMP genes, indicating that the re-introduction of FOXA2 could reverse the phenotype associated with FOXA2 deficiency. Given the important roles of the downregulated and upregulated genes in controlling pancreatic endocrine development and beta-cell functionality, our findings indicate the important role of FOXA2 during pancreatic development and suggest that the patient with *FOXA2* haploinsufficiency may develop diabetes later in life.

RNA-Seq and qPCR analyses showed that the top upregulated genes were associated with neurogenesis and nervous system development, which could be reversed after FOXA2 OE. This result is in agreement with the previous study that showed the activation of neuronal genes in the pancreatic islet of *Foxa2*-deficient mice^11^. Taken together, these findings suggest an important role for FOXA2 in suppressing neuronal fate during pancreatic differentiation to enhance the beta-cell program.

Although *FOXA2* haploinsufficiency led to significant downregulation in the genes related to pancreatic endocrine development, it appears that the expression levels of those genes were still sufficient to generate functional, glucose-responsive beta cells at stage 7. In mice, *Foxa2* haploinsufficiency is enough for normal islet development; however, the deletion of both Foxa1 and Foxa2 in the mouse pancreas leads to pancreatic hypoplasia as a result of a Pdx1 suppression^10^, indicating that Foxa1 can compensate for the absence of Foxa2 in rodents^10^. In this study, we found that *FOXA1* expression was significantly downregulated in PP2 and EPs derived from FOXA2^+/−^ iPSCs. Consistent with our findings, Lee et al. reported the reduction of FOXA1 in the DE and FG derived from FOXA2^−/−^ hESCs^23^. These findings indicate that FOXA1 does not compensate for FOXA2 deficiency, at least, during the early stages of pancreatic development in human, highlighting the physiological differences between mouse and human.

In addition to the significant reduction of INS expression, GCG was significantly downregulated as a result of *FOXA2* haploinsufficiency. Confirming the effect of FOXA2 deficiency on GCG expression, iPSCs lacking FOXA2 generated a very small number of GCG^+^ cells at stage 7. Previous mouse studies showed that Foxa2 activates the expression of Gcg and maintain the alpha cells by transcriptional regulation of *MafB*, *Kir6.2*, *Isl1*, and *Nkx2.2*^20,42,43^. In agreement with these findings, we found that all the endocrine-specific TFs were significantly downregulated as a result of FOXA2 deficiency. These findings indicate that FOXA2 may also be involved in alpha cell development and regulates GCG expression in human pancreatic islet.

Whether the defect in FOXA2 leads to hypo-or hyperglycemia is still controversial. Recent reports have identified two young patients (2 and 5 years old) with hypoglycemic hyperinsulinemic phenotype due to two different *FOXA2* mutations^15,16^. However, a more recent study identified a patient with diabetes due to another *FOXA2* mutation^18^. Of note, these hypo-and hyperglycemic patients shared common features, such as growth hormone deficiency, hypopituitarism, and early hypoglycemia^18^. The hypoglycemic episode followed by hyperglycemia during childhood due to *FOXA2* mutations is similar to some MODY gene mutations such as ABCC8^44^, HNF1A^45^, and HNF4A^46^. The link between FOXA2 defect and diabetes development has been reported in another human genomic study, in which an association of T2D risk alleles with FOXA2-bound enhancers has been found^17^. Taken together, our work supports the concept suggested that FOXA2 defects may lead to hypoglycemia at the early stage of life followed by hyperglycemia during late childhood or at a later stage^18^. Stekelenburg et al^18^. explained the possible mechanism of an early hypoglycemic episode associated with FOXA2 defects as a result of growth hormone deficiency^16^ or the reduction in hepatic gluconeogenesis associated with the downregulation in the GCG expression^18^. Further clinical and experimental studies are needed to understand how *FOXA2* defects influence glucose homeostasis and pancreatic islet functionality over time in human.

In conclusion, our current study provides the first human iPSC model for *FOXA2* haploinsufficiency carrying the genetic information of the patient. Our results indicate that *FOXA2* haploinsufficiency negatively impacted endocrine islet development by downregulating key TFs associated with islet cell development and upregulating genes associated with neuronal development and WNT and BMP pathways. Taken together with other recent reports^17,18,23^, our findings suggest that FOXA2 defects may lead to monogenic diabetes based on the gene dosage. However, it will be of interest to perform further studies to investigate how FOXA2 works with other TFs to main normal pancreatic endocrine development in human.

## Materials and methods

### Study approval

The protocols to obtain blood samples were approved by the Institutional Review Board (IRB) of Sidra Medicine (no. 1702007608) and QBRI/HBKU (no. 2018-002). Informed consent forms were signed by human subjects.

### Generation of iPSCs and their differentiation into pancreatic beta cells

iPSCs were generated from a patient with *FOXA2* heterozygous deletion (QBRIi009-A; FOXA2^+/−^ iPSCs) and healthy controls (Ctr1-iPSCs and Ctr2-iPSCs) as we previously reported^24,25^. All lines were extensively characterized as previously reported^24,25^. The first four stages of the pancreatic differentiation were performed using our protocol^27^ and for further differentiation to beta cells, Rezania et al.^28^ protocol was used (Supplementary Fig. S3A and Supplementary Table S1).

For WNT signaling inhibition, the cells were treated with the WNT inhibitor, 5 µM XAV939 (Torics Bioscience, 3748) for 4 days during stage 4 and the control cells were treated with DMSO. For BMP inhibition, the cells were treated every day during stage 4 with 100 ng/mL NOGGIN, while the control cells were treated with 50 ng/mL of NOGGIN. The cells were collected at end of stage 4 to examine the effect of WNT and BMP inhibition on the dysregulated genes associated with *FOXA2* deficiency.

### Immunocytochemistry, flow cytometry, RT-PCR, and qPCR

Immunostaining and flow cytometry were performed as previously reported^27,29^. RT-PCR and qPCR were performed as previously reported^25^. (The antibody and primer details are listed in protocol was used in Supplementary Tables S2 and S3.)

### Western blot analysis

Total protein was extracted using RIPA buffer and separated by SDS-PAGE. The PVDF membranes were blocked with 10% skim milk in 0.5% TBST, incubated with primary antibodies overnight at 4 °C, and incubated with secondary antibodies at room temperature (protocol was used Supplementary Table S2). The membranes were incubated in SuperSignal West Pico Chemiluminescent substrate (Pierce, Loughborough, UK) detection reagent (VWR) and visualized using the iBright™ CL 1000 Imaging System (Invitrogen).

### RNA-sequencing analysis

The RNA-Sequencing (RNA-Seq) experiments and data analysis were performed as we previously reported^25,47^. For the analysis, the RNA-Seq data were pooled from two Ctr-iPSC lines and three FOXA2^+/−^ iPSC lines of two independent experiments.

### CRISPR/Cas9-mediated generation of FOXA2-KO iPSCs

We generated two *FOXA2* iPSC lines from two control iPSC lines (Ctr1 and Ctr2). The cells were transfected with a plasmid expressing spCas9 and gRNA (Addgene, 79144) using Lipofectamine Stem Transfection Reagent following the manufacturer’s instructions (ThermoFisher Scientific) (Supplementary Fig. S4 and Supplementary Table S8).

### Glucose-stimulated insulin secretion (GSIS)

Beta-cell aggregates were incubated with low (2.8 mM) and high (20 mM) glucose, which was repeated two times. In the end, the clusters were incubated in low glucose with 30 mM KCL to depolarize the cells and release their C-PEP (C-PEPTIDE) contents. The C-PEP concentration in the collected supernatants was measured using Human C-Peptide ELISA Kit (Abcam, ab178641). Each value of C-PEP level of low or high glucose condition was normalized to its own total content of C-PEP in each sample. The stimulation indices were determined through the ratio between the C-PEP level at high glucose concentration to its own basal level secretion at the two challenges.

### Overexpression of FOXA2 and PDX1

At the end of day 2 of stage 4, the cells were dissociated with TrypLE and resuspended in the differentiation medium with 10 μM Y-27632. The resuspended cells were either transfected with FOXA2 plasmid (clone HsCD00330288 in pLenti6.2/v5-DEST; DNASU Plasmid Repository, Arizona State University, Tempe, AZ), PDX1 plasmid (pLenti-GIII-CMV-RFP-2A-Puro, LV259702, abm), or the empty vectors. Transfection was carried out using the Lipofectamine™ 3000 Transfection Reagent following the manufacturer’s instruction (ThermoFisher, L3000015). At 48 h post-transfection (end of stage 4), the cells were harvested for RNA and protein extraction. Some experiments were continued until the end of stage 7 (beta-cell stage) for further analyses.

### Statistical analysis

At least three biological replicates were used in most of the experiments and statistical analysis was carried out using unpaired two-tailed Student’s *t*-test by Prism 8.

## Supplementary information

Supplementary Table 1: Differentiation Media

Supplementary Table 2:Antibody details

Supplementary Table 3: Primer list

Supplementary Table 4: Top downregulated genes in PP2 derived from FOXA2+/- iPSCs in comparison to those derived from Ctr-iPSCs

Supplementary Table 5: Top Upregulated genes in PP2 derived from FOXA2+/- iPSCs in comparison to those derived from Ctr-iPSCs

Supplementary Table 6: Top downregulated genes in the EPs derived from FOXA2+/- iPSCs in comparison to those derived from Ctr-iPSCs

Supplementary Table 7: Top upregulated genes in the EPs derived from FOXA2+/- iPSCs in comparison to those derived from Ctr-iPSCs

Supplementary Table 8: gRNA sequences for FOXA2

Supplementary Figure 1

Supplementary Figure 2

Supplementary Figure 3

Supplementary Figure 4

Supplementary Figure 5

Supplementary Figure 6

## Data Availability

The RNA-Seq data sets generated in the current study are available on the Zenodo repository at https://zenodo.org/record/4362011#.X98RVC0RrAI

## Supplementary information

**Supplementary Information** The online version contains supplementary material available at (10.1038/s41419-021-03390-8).
