# Supplementary material for: Aberrant development of pancreatic beta cells derived from human iPSCs with FOXA2 deficiency
Source: Cell Death Dis. 2021 Jan 20;12(1):103. doi: 10.1038/s41419-021-03390-8 (PMC7817686; doi:10.1038/s41419-021-03390-8)
Supplement: Supplementary file 1 — Supplementary Table 1: Differentiation Media [file 41419_2021_3390_MOESM1_ESM.docx]

**Supplementary Table 1:** Media formulations for pancreatic beta cell differentiation

| **Stage** | **Basal media** | **Cytokines** |
| --- | --- | --- |
| Stage 1  Day 1 | MCDB 131 +  1.5 g/L NaHCO_3_  2 mM glutamax  1% Pen/Strep  0.5% fatty acid free bovine serum albumin | 3 μM CHIR99021  100 ng/mL Activin A  0.25 mM vitamin C  10 μM Y-27632 (Rock inhibitor) |
| Stage 1 Days 2-4 | MCDB 131 +  1.5 g/L NaHCO_3_  2 mM glutamax  1% Pen/Strep  0.5% fatty acid free bovine serum albumin | 100 ng/mL Activin A  0.25 mM vitamin C |
| Stage 2  2 Days | MCDB 131 +  1.5 g/L NaHCO_3_  2 mM glutamax  1% Pen/Strep  0.5% fatty acid-free bovine serum albumin | 50 ng/mL of FGF10  50 ng/mL NOGGIN  3ng/mL Wnt3a  0.25 mM Vitamin C |
| Stage 3  2 Days | DMEM +  4.5g/l glucose  1% Pen/Strep  1% B27 supplement without vitamin A | 2 μM Retinoic acid  50 ng/mL of FGF10  50 ng/mL NOGGIN  0.25 μM SANT-1  0.25 mM Vitamin C |
| Stage 4  4 Days | DMEM +  4.5g/l glucose  1% Pen/Strep  1% B27 supplement without vitamin A | 50 ng/mL NOGGIN  100 ng/mL EGF  10 mM Nicotinamide  0.25 mMVitamin C |
| Stage 5  3 Days | MCDB 131 +  1.5 g/L NaHCO_3_  2 mM glutamax  1% Pen/Strep  1% fatty acid free bovine serum albumin  1:200 Insulin-Transferrin-Selenium-X (ITS) | 0.25 mM Vitamin C  1 μM T3  10 μM ALK 5 inhibitor II  10 μM Zinc sulfate  100 nM LDN193189  0.05 μM Retinoic acid  0.25 μM SANT-1 |
| Stage 6 7 Days | MCDB 131 +  1.5 g/L NaHCO_3_  2 mM glutamax  1% Pen/Strep  2% fatty acid free bovine serum albumin  1:200 Insulin-Transferrin-Selenium-X (ITS) | 0.25 mM Vitamin C  1 μM T3  10 μM ALK 5 inhibitor II  10 μM Zinc sulfate  100 nM LDN193189  100 nM Gamma secretase inhibitor  10 mg/ml Heparin |
| Stage7 7 Days | MCDB 131 +  1.5 g/L NaHCO_3_  2 mM glutamax  1% Pen/Strep  2% fatty acid free bovine serum albumin  1:200 Insulin-Transferrin | 0.25 mM Vitamin C  1 μM T3  10 μM ALK 5 inhibitor II  10 μM Zinc sulfate  100 nM Gamma secretase inhibitor  1 mM N-acetyl cysteine  10 mg/ml Heparin |
