# Supplementary material for: Aberrant development of pancreatic beta cells derived from human iPSCs with FOXA2 deficiency
Source: Cell Death Dis. 2021 Jan 20;12(1):103. doi: 10.1038/s41419-021-03390-8 (PMC7817686; doi:10.1038/s41419-021-03390-8)
Supplement: Supplementary file 2 — Supplementary Table 2:Antibody details [file 41419_2021_3390_MOESM2_ESM.docx]

**Supplementary Table 2:** The details of the antibodies used for immunostaining, flow cytometry, and Western blotting.

| **Antibody** | **Catalog #** | **Company** | **Dilution** |
| --- | --- | --- | --- |
| Anti-FOXA2 | 3143 | CST | WB (1:4000); IS (1:500); FACS (1:100) |
| Anti-SOX17 | CF500096 | OriGene | IS (1:2000); FACS (1:100) |
| Anti-OCT4 | 4286S | CST | IS (1:500); FACS (1:100) |
| Anti-SOX2 | 4900S | CST | IS (1:500) |
| Anti-PDX1 | ab47308 | Abcam | IS (1:1000); FACS (1:100) |
| Anti-NKX6.1 | F55A12 | DSHB | IS (1:2000); FACS (1:100) |
| Anti-NKX2.2 | 74.5A5-c | DSHB | IS (1:1000) |
| Anti-NGN3 | AF3444 | R & D Systems | IS (1:500) |
| Anti-NEUROD1 | ab16508 | Abcam | IS (1:500) |
| Anti-CHGA | MA5-14536 | ThermoFisher Scientific | IS (1:4000) |
| Anti-INSULIN | ab7842 | Abcam | IS (1:1000); FACS (1:100) |
| Anti-C-PEPTIDE | ab8297 | Abcam | IS (1:1000); FACS (1:100) |
| Anti-GLUCAGON | SAB4501137 | Sigma | IS (1:2000); FACS (1:100) |
| Anti-SOMATOSTATIN | A0566 | Dako | IS (1:1000) |
| Anti-NANOG | 9656 | CST | IS (1:500), FACS (1:100) |
| Anti-SSEA4 | 9656 | CST | IS (1:500), FACS (1:100) |
| Anti-TRA-1-60 | 9656 | CST | IS (1:500), FACS (1:100) |
| Anti-TRA-81 | 9656 | CST | IS (1:500), FACS (1:100) |
| Anti-NESTIN | MAB1259 | R & D Systems | IS (1:500) |
| Anti-BRACHYURY | 140661 | Abcam | IS (1:1000) |
| anti-β-Actin | sc-47778 | Santa Cruz | WB (1:10000) |
| Alexa Fluor 488 anti-rabbit IgG | A-21206 | ThermoFisher Scientific | IS (1:500), FACS (1:300) |
| Alexa Fluor 568 anti-rabbit IgG | A-10042 | ThermoFisher Scientific | IS (1:500) |
| Alexa Fluor 568 anti-mouse IgG | A-10037 | ThermoFisher Scientific | IS (1:500) |
| Alexa Fluor 488 anti-mouse IgG | A-21202 | ThermoFisher Scientific | IS (1:500), FACS (1:300) |
| Alexa Fluor 647 anti-mouse IgG | A31571 | ThermoFisher Scientific | FACS (1:300) |
| Alexa Fluor 488 anti-sheep IgG | A11015 | ThermoFisher Scientific | IS (1:500) |
| Alexa Fluor 488 anti-guinea pig IgG | A11073 | ThermoFisher Scientific | IS (1:500), FACS (1:300) |
| Peroxidase AffiniPure Donkey anti-Rabbit IgG (H+L) | 711-035-152 | Jackson ImmunoResearch Laboratories | WB (1:10000) |
| Peroxidase AffiniPure Donkey anti-MouseIgG (H+L) | 715-035-150 | Jackson ImmunoResearch Laboratories | WB (1:10000) |
