# Supplementary material for: Aberrant development of pancreatic beta cells derived from human iPSCs with FOXA2 deficiency
Source: Cell Death Dis. 2021 Jan 20;12(1):103. doi: 10.1038/s41419-021-03390-8 (PMC7817686; doi:10.1038/s41419-021-03390-8)
Supplement: Supplementary file 3 — Supplementary Table 3: Primer list [file 41419_2021_3390_MOESM3_ESM.docx]

**Supplementary Table 3:** The list of primers used for Real-time PCR (qPCR)

| **Gene Name** | **Forward** | **Reverse** | **Product size** |
| --- | --- | --- | --- |
| GAPDH | ACGACCACTTTGTCAAGCTCATTTC | GCAGTGAGGGTCTCTCTCTTCCTCT | 132 |
| FOXA2 | GGGAGCGGTGAAGATGGA | TCATGTTGCTCACGGAGGAGTA | 89 |
| PDX1 | CGTCCAGCTGCCTTTCCCAT | CCGTGAGATGTACTTGTTGAATAGGA | 178 |
| NKX6.1 | GCCCGCCCTGGAGGGACGCA | ACGAATAGGCCAAACGAGCCC | 186 |
| NKX2.2 | AAACCATGTCACGCGCTCA | GGCGTTGTACTGCATGTGCT | 111 |
| NEUROD1 | GCCCCAGGGTTATGAGACTAT | GAGAACTGAGACACTCGTCTGT | 146 |
| INSULIN | AAGAGGCCATCAAGCAGATCA | CAGGAGGCGCATCCACA | 58 |
| NEUROG3 | CCTTACCCTTAGCACCA | CCCTCTACTCCCCAGTCTCC | 176 |
| HNF6 | GGACCTCAAGATAGCAGGTTTAT | CAGAATGCAGGTGAGCTAAGT | 99 |
| SOX17 | GCATTCTGGAATGAGCCTACT | GGGCAGGTCAAGCTTATGAT | 146 |
| GLUCAGON | CTCTTCACCTGCTCTGTTCTAC | TGGATTTCTCCTCTGTGTCTTG | 138 |
| PTF1A | ATTATGGCCTCCCTCCCCTA | AGTTTTCTGGGGTCCTCTGG | 118 |
| HNF1-beta | ACACACCTCCCATCCTCAAG | CATTTTAGCAGCCCTCCAAG | 113 |
| GATA6 | AAGCGCGTGCCTTCATCA | TCATAGCAAGTGGTCTGGGC | 157 |
| FOXA1 | GCAATACTCGCCTTACGGCT | GGCGTACTACCAAGGTGTGTA | 120 |
| ABCC8 | TTGCCGAAACCGTAGAAGG | CGGTTCCAGCAGAAGCTTC | 72 |
| AMYLASE | CGTTGGCCAAGACAGTTTCA | GCCATCGATGTTCACAGACC | 139 |
| HHEX | GCGAGAGACAGGTCAAAACC | AGGGCGAACATTGAGAGCTA | 186 |
| HNF4A | CCAAGTACATCCCAGCTTTC | TTGGCATCTGGGTCAAAG | 295 |
| INSR | AATTGGGAACTACTCCTTCT | CCTGAAACTTCTTCCATCTT | 159 |
| KCNJ11 | GCGCTTTGTGCCCATTGTA | TTGACGGTGTTGCCAAACTTG | 75 |
| GATA4 | GCCTCCTCTGCCTGGTAAT | CAGTCCCATCAGCGTGTAAA | 120 |
| PTPRN | CCTACCAAGCAGAGCCAAAC3 | TGGTCATAGGGCAGGAAGTC | 91 |
| ADRA2A | CTTCTGGTTCGGCTACTGCAAC | GGAAACCTCACACGATCCGCTT | 128 |
| NOTCH1 | CTGGTCAGGGAAATCGTG | TGGGCAGTGGCAGATGTAG | 106 |
| HES1 | AGTGAAGCACCTCCGGAAC | TCACCTCGTTCATGCACTC | 113 |
| MNX1 | CGAGACCCAGGTGAAGATTT | CTTCTGTTTCTCCGCTTCCT | 100 |
| PROX1 | AAAGTCAAATGTACTCCGCAAGC | CTGGGAAATTATGGTTGCTCCT | 91 |
| DLX5 | CGCCTCGCTGGGATTG | CTTGATCTTGGATCTTTTGTTCTGAA | 67 |
| OTX1 | CTAGAGTCCAGGTCTGGTT | CTGGAGAGGACTTCTTCTTG | 105 |
| BMP4 | GACTACATGCGGGATCTTTAC | GGATGTTCTCCAGATGTTCTTC | 148 |
| DKK1 | CAATTCCAACGCTATCAAGAAC | GGGTACGGCTGGTAGTT | 141 |
| MSX2 | TTACCACATCCCAGCTCCTC | CCTGGGTCTCTGTGAGGTTC | 111 |
| NOG | TAGCTTTCTGGTTCCTGTAATG | ACAGTAGAAGCCGGTAACT | 131 |
| MSX1 | CTCGTCAAAGCCGAGAGC | CGGTTCGTCTTGTGTTTGC | 128 |
| EGR2 | AACGGAGTGGCCGGAGAT | ATGGGAGATCCAACGACCTCTT | 70 |
| EMX2 | GCTTCTAAGGCTGGAACACG | TTGCGAATCTGAGCCTTCTT | 172 |
| DLX2 | CACCCAGACTCAGGTCAAA | GAAGCACAAGGTGGAGAAG | 123 |
| OTX2 | GCTGTAAGTTCCACTGCTC | GCTGTAAGTTCCACTGCTC | 135 |
| CDX2 | CTGGAGCTGGAGAAGGAGTTTC | ATTTTAACCTGCCTCTCAGAGAGC | 101 |
