# Supplementary material for: Aberrant development of pancreatic beta cells derived from human iPSCs with FOXA2 deficiency
Source: Cell Death Dis. 2021 Jan 20;12(1):103. doi: 10.1038/s41419-021-03390-8 (PMC7817686; doi:10.1038/s41419-021-03390-8)
Supplement: Supplementary file 4 — Supplementary Table 4: Top downregulated genes in PP2 derived from FOXA2+/- iPSCs in comparison to those derived from Ctr-iPSCs [file 41419_2021_3390_MOESM4_ESM.docx]

**Supplementary Table 4:** Top downregulated genes in pancreatic progenitors (PP2) derived from FOXA2^+/-^ iPSCs in comparison to those derived from Ctr-iPSCs (*p*<0.05).

| **Gene Name** | **Gene symbol** | **Log2-FC** | **P-value** |
| --- | --- | --- | --- |
| Glucagon | GCG | -6.6452 | 0.00005 |
| NK6 homeobox 1 | NKX6-1 | -6.14941 | 0.00005 |
| Pancreas associated transcription factor 1a | PTF1A | -5.33184 | 0.00005 |
| Neurogenin 3 | NEUROG3 | -5.08149 | 0.00015 |
| NK2 homeobox 2 | NKX2-2 | -4.92869 | 0.00005 |
| Protein tyrosine phosphatase receptor type N | PTPRN | -4.8877 | 0.00005 |
| Neuronal differentiation 1 | NEUROD1 | -4.12469 | 0.00005 |
| Retinol binding protein 4 | RBP4 | -4.05273 | 0.00005 |
| Nuclear receptor subfamily 5 group A member 2 | NR5A2 | -3.92367 | 0.00005 |
| Adrenoceptor alpha 2A | ADRA2A | -3.77831 | 0.00005 |
| Fibrinogen alpha chain | FGA | -3.75132 | 0.00005 |
| Pancreatic and duodenal homeobox 1 | PDX1 | -3.7346 | 0.00005 |
| MLX interacting protein like | MLXIPL | -3.73119 | 0.00005 |
| Paired box 4 | PAX4 | -3.67416 | 0.0001 |
| Gastric inhibitory polypeptide receptor | GIPR | -3.57431 | 0.00005 |
| hes related family bHLH transcription factor with YRPW motif 1 | HEY1 | -3.26291 | 0.00005 |
| Proopiomelanocortin | POMC | -3.25985 | 0.00005 |
| Fibrinogen gamma chain | FGG | -3.13437 | 0.00005 |
| Forkhead box A2 | FOXA2 | -2.89118 | 0.00005 |
| Forkhead box A3 | FOXA3 | -2.81959 | 0.00005 |
| Hes related family bHLH transcription factor with YRPW motif like | HEYL | -2.77587 | 0.00005 |
| SRY-box transcription factor 9 | SOX9 | -2.76177 | 0.00005 |
| Hepatocyte nuclear factor 4 alpha | HNF4A | -2.7028 | 0.00005 |
| Annexin A4 | ANXA4 | -2.66599 | 0.00005 |
| INSM transcriptional repressor 1 | INSM1 | -2.59546 | 0.00005 |
| Glutamate receptor interacting protein 2 | GRIP2 | -2.47909 | 0.00005 |
| RAB26, member RAS oncogene family | RAB26 | -2.44905 | 0.00005 |
| CCAAT enhancer binding protein alpha | CEBPA | -2.20028 | 0.00005 |
| One cut homeobox 1 | ONECUT1 | -2.11407 | 0.00005 |
| Motor neuron and pancreas homeobox 1 | MNX1 | -2.10338 | 0.00005 |
| Fibroblast growth factor receptor 4 | FGFR4 | -2.06406 | 0.00005 |
| Prospero homeobox 1 | PROX1 | -1.89287 | 0.00005 |
| GATA binding protein 6 | GATA6 | -1.88623 | 0.00005 |
| Fibrinogen beta chain | FGB | -1.81672 | 0.00005 |
| Mastermind like transcriptional coactivator 3 | MAML3 | -1.56053 | 0.00005 |
| Notch receptor 1 | NOTCH1 | -1.5149 | 0.00005 |
| Quinoid dihydropteridine reductase | QDPR | -1.4669 | 0.00005 |
| RAB3B, member RAS oncogene family | RAB3B | -1.4531 | 0.00005 |
| Transcription factor 7 like 2 | TCF7L2 | -1.44541 | 0.00005 |
| HNF1 homeobox A | HNF1A | -1.38754 | 0.00035 |
| Jagged canonical Notch ligand 1 | JAG1 | -1.37511 | 0.00005 |
| Hes family bHLH transcription factor 1 | HES1 | -1.37068 | 0.00005 |
| Hematopoietically expressed homeobox | HHEX | -1.26814 | 0.00005 |
| Insulin receptor | INSR | -1.25574 | 0.00005 |
| LLGL scribble cell polarity complex component 2 | LLGL2 | -1.24872 | 0.00005 |
| Syntaxin binding protein 5 | STXBP5 | -1.20295 | 0.00005 |
| Exophilin 5 | EXPH5 | -1.14012 | 0.00005 |
| RAB3D | RAB3D | -1.12408 | 0.00005 |
| Phospholipid scramblase 3 | PLSCR3 | -1.00196 | 0.00295 |
