# Supplementary material for: Aberrant development of pancreatic beta cells derived from human iPSCs with FOXA2 deficiency
Source: Cell Death Dis. 2021 Jan 20;12(1):103. doi: 10.1038/s41419-021-03390-8 (PMC7817686; doi:10.1038/s41419-021-03390-8)
Supplement: Supplementary file 5 — Supplementary Table 5: Top Upregulated genes in PP2 derived from FOXA2+/- iPSCs in comparison to those derived from Ctr-iPSCs [file 41419_2021_3390_MOESM5_ESM.docx]

**Supplementary Table 5:** Top Upregulated genes in pancreatic progenitors (PP2) derived from FOXA2^+/-^ iPSCs in comparison to those derived from Ctr-iPSCs (*p*<0.05).

| **Gene Name** | **Gene symbol** | **Log2-FC** | **P-value** |
| --- | --- | --- | --- |
| Early growth response 2 | EGR2 | 5.09581 | 0.00005 |
| Distal-less homeobox 5 | DLX5 | 4.8667 | 0.00005 |
| Empty spiracles homeobox 2 | EMX2 | 4.50598 | 0.0092 |
| Distal-less homeobox 2 | DLX2 | 4.20067 | 0.0156 |
| SLIT and NTRK like family member 1 | SLITRK1 | 4.15556 | 0.00005 |
| FEZ family zinc finger 1 | FEZF1 | 4.09393 | 0.00005 |
| Wnt family member 1 | WNT1 | 3.85421 | 0.00005 |
| Orthodenticle homeobox 1 | OTX1 | 3.83476 | 0.00005 |
| Maternally expressed 3 | MEG3 | 3.60831 | 0.00005 |
| BarH like homeobox 1 | BARHL1 | 3.60246 | 0.0001 |
| POU class 3 homeobox 3 | POU3F3 | 3.58626 | 0.00015 |
| Protocadherin 18 | PCDH18 | 3.55667 | 0.00005 |
| Leucine rich glioma inactivated 1 | LGI1 | 3.53882 | 0.00005 |
| Ventral anterior homeobox 1 | VAX1 | 3.51031 | 0.00005 |
| POU class 3 homeobox 2 | POU3F2 | 3.46876 | 0.00005 |
| Pleiotrophin | PTN | 3.38703 | 0.00005 |
| Transcription factor AP-2 alpha | TFAP2A | 3.38223 | 0.00005 |
| FEZ family zinc finger 2 | FEZF2 | 3.38214 | 0.00005 |
| EPH receptor A7 | EPHA7 | 3.32057 | 0.00005 |
| Homeobox B1 | HOXB1 | 3.26829 | 0.00015 |
| Iroquois homeobox 3 | IRX3 | 3.26346 | 0.00005 |
| Proteolipid protein 1 | PLP1 | 3.05474 | 0.00055 |
| Homeobox B2 | HOXB2 | 3.03702 | 0.00005 |
| Paired box 3 | PAX3 | 2.98174 | 0.00005 |
| Dickkopf WNT signaling pathway inhibitor 1 | DKK1 | 2.95475 | 0.00005 |
| Secreted frizzled related protein 2 | SFRP2 | 2.90006 | 0.00005 |
| Wnt family member 8B | WNT8B | 2.82475 | 0.00005 |
| Hyaluronan and proteoglycan link protein 1 | HAPLN1 | 2.81144 | 0.00005 |
| Protein tyrosine phosphatase receptor type Z1 | PTPRZ1 | 2.79304 | 0.00005 |
| GLI family zinc finger 3 | GLI3 | 2.78548 | 0.00005 |
| Orthodenticle homeobox 2 | OTX2 | 2.75488 | 0.00005 |
| SLIT and NTRK like family member 5 | SLITRK5 | 2.72251 | 0.00005 |
| SIX homeobox 3 | SIX3 | 2.66304 | 0.00005 |
| Sphingosine-1-phosphate receptor 1 | S1PR1 | 2.64875 | 0.00005 |
| Solute carrier family 7 member 11 | SLC7A11 | 2.62824 | 0.00005 |
| Bone morphogenetic protein 4 | BMP4 | 2.55465 | 0.00005 |
| Heart and neural crest derivatives expressed 1 | HAND1 | 2.55215 | 0.00005 |
| Neurogenin 1 | NEUROG1 | 2.50008 | 0.0143 |
| SRY-box transcription factor 2 | SOX2 | 2.49972 | 0.00005 |
| Wnt family member 5A | WNT5A | 2.49486 | 0.00005 |
| Axin 2 | AXIN2 | 2.46106 | 0.00005 |
| Frizzled class receptor 10 | FZD10 | 2.45938 | 0.00005 |
| Frizzled class receptor 3 | FZD3 | 2.38824 | 0.00005 |
| HESX homeobox 1 | HESX1 | 2.36166 | 0.0072 |
| Inhibitor of DNA binding 1, HLH protein | ID1 | 2.28563 | 0.00005 |
| Inhibitor of DNA binding 3, HLH protein | ID3 | 2.25986 | 0.00005 |
| Paired box 6 | PAX6 | 2.20386 | 0.00005 |
| Zic family member 3 | ZIC3 | 2.19123 | 0.00005 |
| Frizzled class receptor 6 | FZD6 | 2.15747 | 0.00005 |
| Frizzled class receptor 1 | FZD1 | 2.10681 | 0.00005 |
| APC regulator of WNT signaling pathway 2 | APC2 | 1.94335 | 0.00005 |
| SRY-box transcription factor 17 | SOX17 | 1.77053 | 0.00005 |
| Sclerostin domain containing 1 | SOSTDC1 | 1.76281 | 0.00035 |
| APC down-regulated 1 | APCDD1 | 1.76159 | 0.00005 |
| Snail family transcriptional repressor 2 | SNAI2 | 1.52373 | 0.00005 |
| Dishevelled binding antagonist of beta catenin 1 | DACT1 | 1.49508 | 0.00005 |
| Carboxypeptidase Z | CPZ | 1.49414 | 0.00005 |
| Cerberus 1, DAN family BMP antagonist | CER1 | 1.49248 | 0.00005 |
| Noggin | NOG | 1.44392 | 0.00215 |
| Dorsal inhibitory axon guidance protein | DRAXIN | 1.39975 | 0.00005 |
| TLE family member 4 | TLE4 | 1.34429 | 0.00005 |
| Collagen triple helix repeat containing 1 | CTHRC1 | 1.3423 | 0.00025 |
| Insulin like growth factor binding protein 4 | IGFBP4 | 1.32762 | 0.00005 |
| Secreted frizzled related protein 1 | SFRP1 | 1.27746 | 0.00005 |
| Shisa family member 2 | SHISA2 | 1.22871 | 0.00005 |
| Msh homeobox 2 | MSX2 | 1.20335 | 0.00005 |
| Msh homeobox 1 | MSX1 | 1.1307 | 0.00005 |
| Repulsive guidance molecule BMP co-receptor a | RGMA | 1.1274 | 0.00005 |
| Repulsive guidance molecule BMP co-receptor b | RGMB | 1.06993 | 0.00005 |
| Crumbs cell polarity complex component 2 | CRB2 | 1.06132 | 0.00005 |
