# Supplementary material for: Aberrant development of pancreatic beta cells derived from human iPSCs with FOXA2 deficiency
Source: Cell Death Dis. 2021 Jan 20;12(1):103. doi: 10.1038/s41419-021-03390-8 (PMC7817686; doi:10.1038/s41419-021-03390-8)
Supplement: Supplementary file 6 — Supplementary Table 6: Top downregulated genes in the EPs derived from FOXA2+/- iPSCs in comparison to those derived from Ctr-iPSCs [file 41419_2021_3390_MOESM6_ESM.docx]

**Supplementary Table 6:** Top downregulated genes in pancreatic endocrine progenitors (EPs) derived from FOXA2^+/-^ iPSCs in comparison to those derived from Ctr-iPSCs (*p*<0.05).

| **Gene Name** | **Gene symbol** | **Log2-FC** | **P-value** |
| --- | --- | --- | --- |
| CF transmembrane conductance regulator | CFTR | -3.902566 | 0.00005 |
| NK6 homeobox 1 | NKX6-1 | -3.5153792 | 0.00005 |
| Carboxypeptidase A2 | CPA2 | -3.5022496 | 0.00005 |
| G protein-coupled receptor 119 | GPR119 | -3.4131894 | 0.00005 |
| Adenylate cyclase activating polypeptide 1 | ADCYAP1 | -3.2843522 | 0.00005 |
| Free fatty acid receptor 1 | FFAR1 | -3.20245 | 0.0001 |
| Glucagon | GCG | -3.1514541 | 0.00005 |
| Neuronal differentiation 1 | NEUROD1 | -2.9654337 | 0.00005 |
| Nuclear receptor subfamily 5 group A member 2 | NR5A2 | -2.944397 | 0.00005 |
| Solute carrier family 4 member 4 | SLC4A4 | -2.9259283 | 0.00005 |
| Annexin A4 | ANXA4 | -2.9144769 | 0.00005 |
| Neurogenin 3 | NEUROG3 | -2.7768276 | 0.00005 |
| Adrenoceptor alpha 2A | ADRA2A | -2.7139252 | 0.00005 |
| Nuclear receptor subfamily 0 group B member 2 | NR0B2 | -2.7073344 | 0.00005 |
| Pancreatic and duodenal homeobox 1 | PDX1 | -2.6609536 | 0.00005 |
| NK2 homeobox 2 | NKX2-2 | -2.624521 | 0.00005 |
| ChaC glutathione specific gamma glutamylcyclotransferase 1 | CHAC1 | -2.4079046 | 0.00005 |
| Forkhead box A2 | FOXA2 | -2.2755078 | 0.00005 |
| Bone morphogenetic protein 2 | BMP2 | -2.1034274 | 0.00005 |
| One cut homeobox 1 | ONECUT1 | -2.0906039 | 0.00005 |
| ATPase sarcoplasmic/endoplasmic reticulum Ca2+ transporting 3 | ATP2A3 | -2.0625371 | 0.00005 |
| Protein tyrosine phosphatase receptor type N | PTPRN | -2.0366628 | 0.00005 |
| Prospero homeobox 1 | PROX1 | -1.9509034 | 0.00005 |
| Gastric inhibitory polypeptide receptor | GIPR | -1.8778349 | 0.00005 |
| Neuralized E3 ubiquitin protein ligase 1 | NEURL1 | -1.8643932 | 0.00005 |
| NK6 homeobox 2 | NKX6-2 | -1.8013399 | 0.00005 |
| GATA binding protein 6 | GATA6 | -1.7575668 | 0.00005 |
| Carboxypeptidase A1 | CPA1 | -1.6945314 | 0.00005 |
| Pancreas associated transcription factor 1a | PTF1A | -1.6234256 | 0.00005 |
| Motor neuron and pancreas homeobox 1 | MNX1 | -1.6037003 | 0.00005 |
| GATA binding protein 4 | GATA4 | -1.6006882 | 0.00005 |
| Secretin receptor | SCTR | -1.5912382 | 0.00005 |
| Cadherin 6 | CDH6 | -1.5711442 | 0.00005 |
| Hematopoietically expressed homeobox | HHEX | -1.5491027 | 0.00005 |
| Transforming growth factor beta receptor 2 | TGFBR2 | -1.5071366 | 0.00005 |
| SRY-box transcription factor 9 | SOX9 | -1.4724065 | 0.00005 |
| ISL LIM homeobox 1 | ISL1 | -1.3432276 | 0.00005 |
| G protein-coupled receptor class C group 5 member B | GPRC5B | -1.3394475 | 0.00005 |
| Forkhead box A3 | FOXA3 | -1.2851889 | 0.00005 |
| ATPase plasma membrane Ca2+ transporting 4 | ATP2B4 | -1.2405588 | 0.00005 |
| Inositol 1,4,5-trisphosphate receptor type 3 | ITPR3 | -1.2216779 | 0.00005 |
| Aph-1 homolog B, gamma-secretase subunit | APH1B | -1.1040607 | 0.00005 |
| Jagged canonical Notch ligand 1 | JAG1 | -1.0969859 | 0.00005 |
| p53 apoptosis effector related to PMP22 | PERP | -1.0886279 | 0.00005 |
| ATPase Na+/K+ transporting subunit alpha 1 | ATP1A1 | -1.049188 | 0.00005 |
| Endoplasmic reticulum oxidoreductase 1 beta | ERO1B | -1.0424405 | 0.00005 |
| X-box binding protein 1 | XBP1 | -1.0021166 | 0.00005 |
