# Supplementary material for: Aberrant development of pancreatic beta cells derived from human iPSCs with FOXA2 deficiency
Source: Cell Death Dis. 2021 Jan 20;12(1):103. doi: 10.1038/s41419-021-03390-8 (PMC7817686; doi:10.1038/s41419-021-03390-8)
Supplement: Supplementary file 7 — Supplementary Table 7: Top upregulated genes in the EPs derived from FOXA2+/- iPSCs in comparison to those derived from Ctr-iPSCs [file 41419_2021_3390_MOESM7_ESM.docx]

**Supplementary Table 7:** Top upregulated genes in the pancreatic endocrine progenitors (EPs) derived from FOXA2^+/-^ iPSCs in comparison to those derived from Ctr-iPSCs.

| **Gene Name** | **Gene symbol** | **Log2-FC** | **P-value** |
| --- | --- | --- | --- |
| Retina and anterior neural fold homeobox | RAX | 9.35428128 | 0.0102 |
| LIM homeobox 2 | LHX2 | 8.20050441 | 0.0001 |
| FEZ family zinc finger 2 | FEZF2 | 7.93132528 | 0.00405 |
| Forkhead box G1 | FOXG1 | 7.61159458 | 0.00005 |
| Empty spiracles homeobox 2 | EMX2 | 7.21345635 | 0.00005 |
| Ventral anterior homeobox 1 | VAX1 | 7.19629823 | 0.0075 |
| FEZ family zinc finger 1 | FEZF1 | 7.09427012 | 0.0003 |
| Nuclear receptor subfamily 2 group E member 1 | NR2E1 | 6.94751052 | 0.0165 |
| Fibroblast growth factor 8 | FGF8 | 6.60072344 | 0.00495 |
| BarH like homeobox 1 | BARHL1 | 6.22760956 | 0.00005 |
| SIX homeobox 3 | SIX3 | 6.11607371 | 0.00005 |
| NK2 homeobox 1 | NKX2-1 | 6.09606157 | 0.01365 |
| DMRT like family A2 | DMRTA2 | 5.76240875 | 0.0008 |
| Orthodenticle homeobox 2 | OTX2 | 5.68651215 | 0.00005 |
| POU class 3 homeobox 1 | POU3F1 | 5.56447876 | 0.00005 |
| Distal-less homeobox 6 | DLX6 | 5.37715532 | 0.00025 |
| Homeobox B1 | HOXB1 | 5.35116487 | 0.0002 |
| Paired like homeobox 2B | PHOX2B | 5.0674617 | 0.0075 |
| Orthodenticle homeobox 1 | OTX1 | 4.95642341 | 0.00005 |
| Zic family member 1 | ZIC1 | 4.89648909 | 0.00005 |
| BARX homeobox 1 | BARX1 | 4.8589217 | 0.0001 |
| Protein tyrosine phosphatase receptor type Z1 | PTPRZ1 | 4.6766046 | 0.00005 |
| Neuritin 1 | NRN1 | 4.5686171 | 0.00005 |
| Forkhead box B1 | FOXB1 | 4.53783606 | 0.01635 |
| Zic family member 5 | ZIC5 | 4.46211326 | 0.00005 |
| Homeobox D3 | HOXD3 | 4.00988918 | 0.00005 |
| Secreted frizzled related protein 2 | SFRP2 | 3.94886128 | 0.00005 |
| SRY-box transcription factor 2 | SOX2 | 3.88503346 | 0.00005 |
| Wnt family member 7B | WNT7B | 3.84749943 | 0.00005 |
| Cytochrome P450 family 26 subfamily C member 1 | CYP26C1 | 3.83130817 | 0.00005 |
| Distal-less homeobox 5 | DLX5 | 3.69837885 | 0.00005 |
| empty spiracles homeobox 1 | EMX1 | 3.53943692 | 0.00005 |
| Maternally expressed 3 | MEG3 | 3.53333659 | 0.00005 |
| Neurogenin 1 | NEUROG1 | 3.41513621 | 0.0134 |
| Noggin | NOG | 3.10390936 | 0.00005 |
| GLI family zinc finger 3 | GLI3 | 3.07537878 | 0.00005 |
| Shisa family member 2 | SHISA2 | 2.96527784 | 0.00005 |
| APC membrane recruitment protein 2 | AMER2 | 2.92905645 | 0.00005 |
| Wnt family member 1 | WNT1 | 2.6568279 | 0.00005 |
| Wnt family member 5B | WNT5B | 2.63948823 | 0.00005 |
| Wnt family member 5A | WNT5A | 2.43892285 | 0.00005 |
| TLE family member 4 | TLE4 | 2.32436225 | 0.00005 |
| Dishevelled binding antagonist of beta catenin 3 | DACT3 | 2.14149889 | 0.00005 |
| Carboxypeptidase Z | CPZ | 2.05474506 | 0.00005 |
| Secreted frizzled related protein 1 | SFRP1 | 1.93160094 | 0.00005 |
| Insulin like growth factor binding protein 4 | IGFBP4 | 1.87605237 | 0.00005 |
| Frizzled class receptor 2 | FZD2 | 1.5163281 | 0.00005 |
| Transmembrane protein 88 | TMEM88 | 1.42801657 | 0.00005 |
| Frizzled class receptor 1 | FZD1 | 1.36295171 | 0.00005 |
| Cadherin EGF LAG seven-pass G-type receptor 2 | CELSR2 | 1.35353148 | 0.00005 |
| Frizzled class receptor 3 | FZD3 | 1.30583511 | 0.00005 |
| Sclerostin domain containing 1 | SOSTDC1 | 1.23330111 | 0.00005 |
| Glypican 3 | GPC3 | 1.16254591 | 0.00005 |
| HMG-box containing 4 | HMGXB4 | 1.05948012 | 0.00005 |
| Chordin | CHRD | 1.0050554 | 0.0001 |
