# Supplementary material for: Aberrant development of pancreatic beta cells derived from human iPSCs with FOXA2 deficiency
Source: Cell Death Dis. 2021 Jan 20;12(1):103. doi: 10.1038/s41419-021-03390-8 (PMC7817686; doi:10.1038/s41419-021-03390-8)
Supplement: Supplementary file 8 — Supplementary Table 8: gRNA sequences for FOXA2 [file 41419_2021_3390_MOESM8_ESM.docx]

**Supplementary Table 8:** gRNA sequences for FOXA2 targeting, and primer sequences for PCR genotyping.

| **Name** | **Sequence** | **Annotation** |
| --- | --- | --- |
| FOXA2 gRNA1 | CACCGTAGTAGCTGCTCCAGTCGGA | gRNA sequence (5’of PAM): + strand |
| FOXA2 gRNA2 | CACCGGGCTCGGGCAACATGAGCGC | gRNA sequence (5’of PAM): + strand |
| FOXA2_F1 | atataagcttTCAAGGCTAACAGTGTGTCG | Forward primer for PCR genotyping (5’ to 3’) |
| FOXA2_R1 | atatgaattcGGGTGATTGCTGGTCGTTTG | Reverse primer for PCR genotyping (5’ to 3’) |
| FOXA2_F2 | atataagcttGGAGTTCATGTTGGCGTAGG | Forward primer for PCR genotyping (5’ to 3’) |
| FOXA2_R2 | atatgaattcAGCCGCGGTGCTAACGCTGTC | Reverse primer for PCR genotyping (5’ to 3’) |
