# Supplementary figures and images for: Aberrant development of pancreatic beta cells derived from human iPSCs with FOXA2 deficiency
Source: Cell Death Dis. 2021 Jan 20;12(1):103. doi: 10.1038/s41419-021-03390-8 (PMC7817686; doi:10.1038/s41419-021-03390-8)

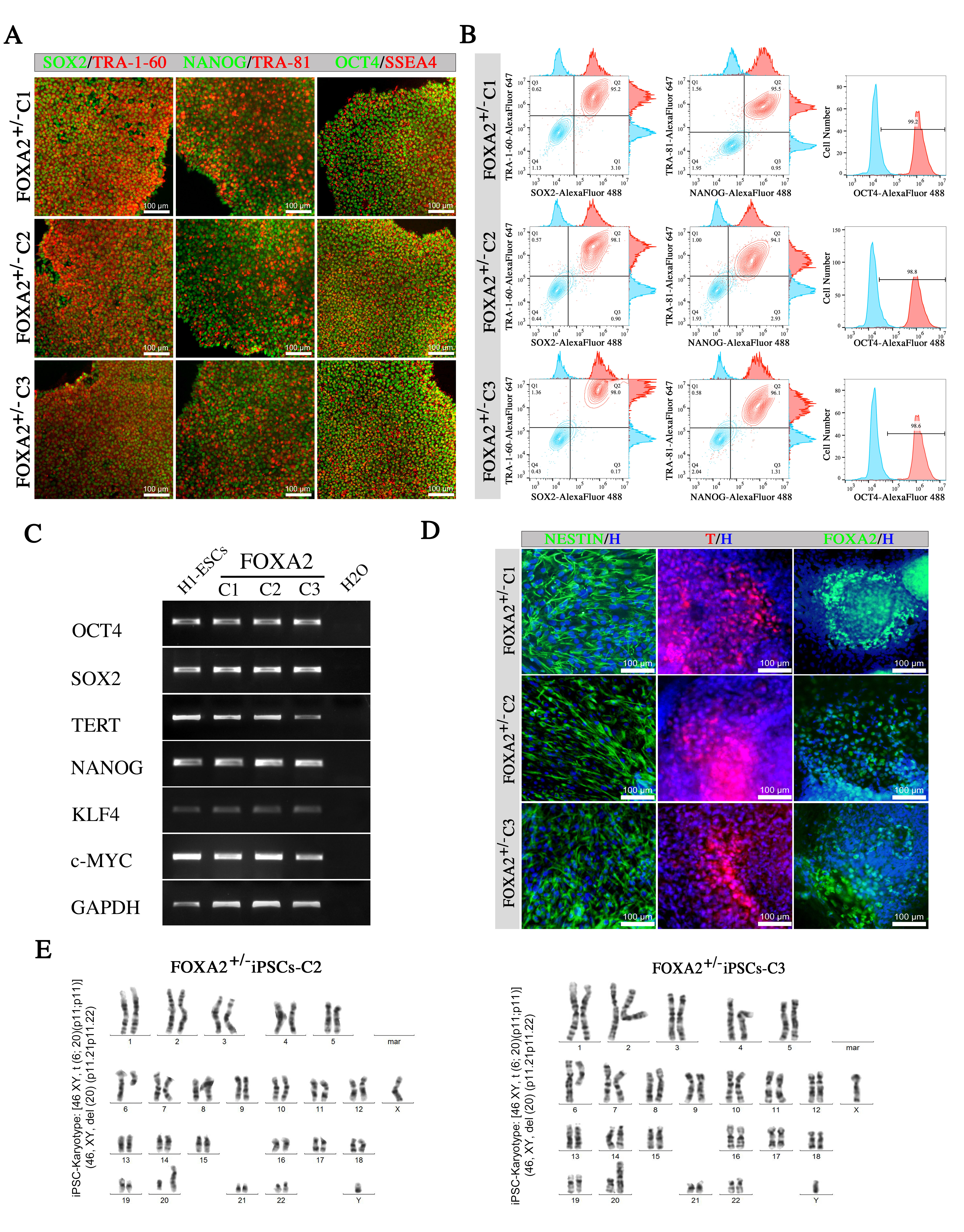

Supplement: Supplementary file 9 — Supplementary Figure 1 [file 41419_2021_3390_MOESM9_ESM.jpg]

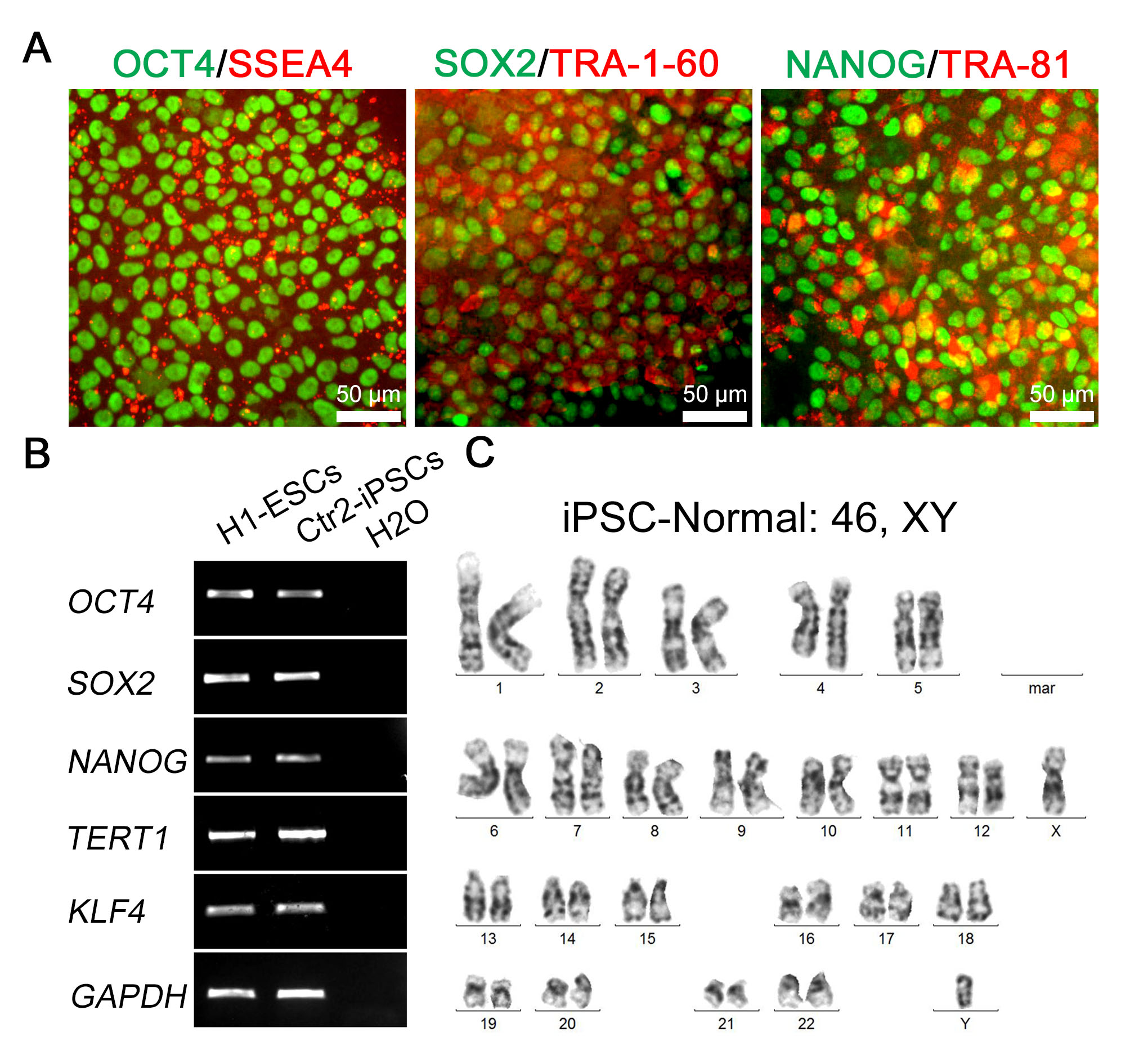

Supplement: Supplementary file 10 — Supplementary Figure 2 [file 41419_2021_3390_MOESM10_ESM.jpg]

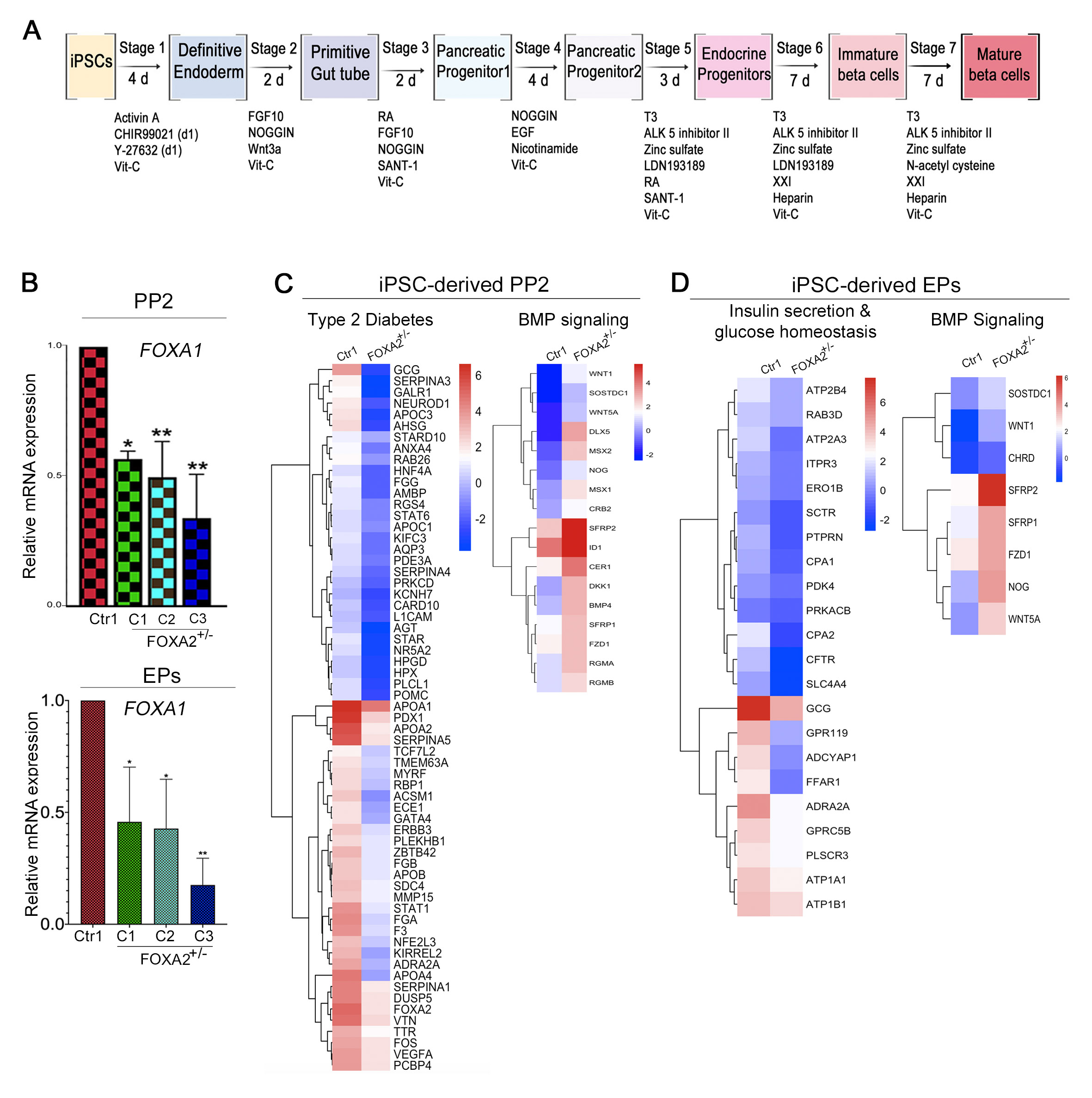

Supplement: Supplementary file 11 — Supplementary Figure 3 [file 41419_2021_3390_MOESM11_ESM.jpg]

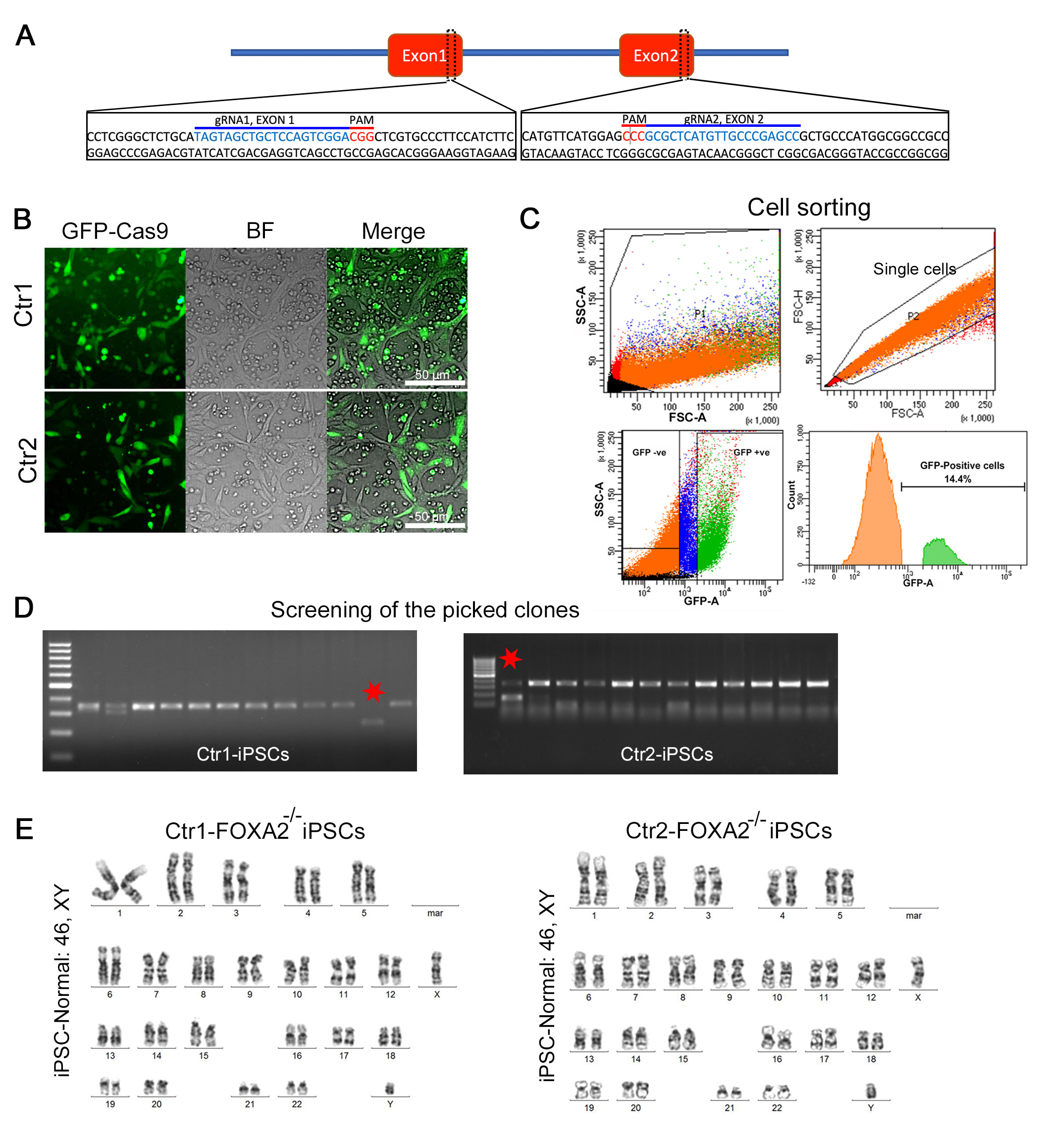

Supplement: Supplementary file 12 — Supplementary Figure 4 [file 41419_2021_3390_MOESM12_ESM.jpg]

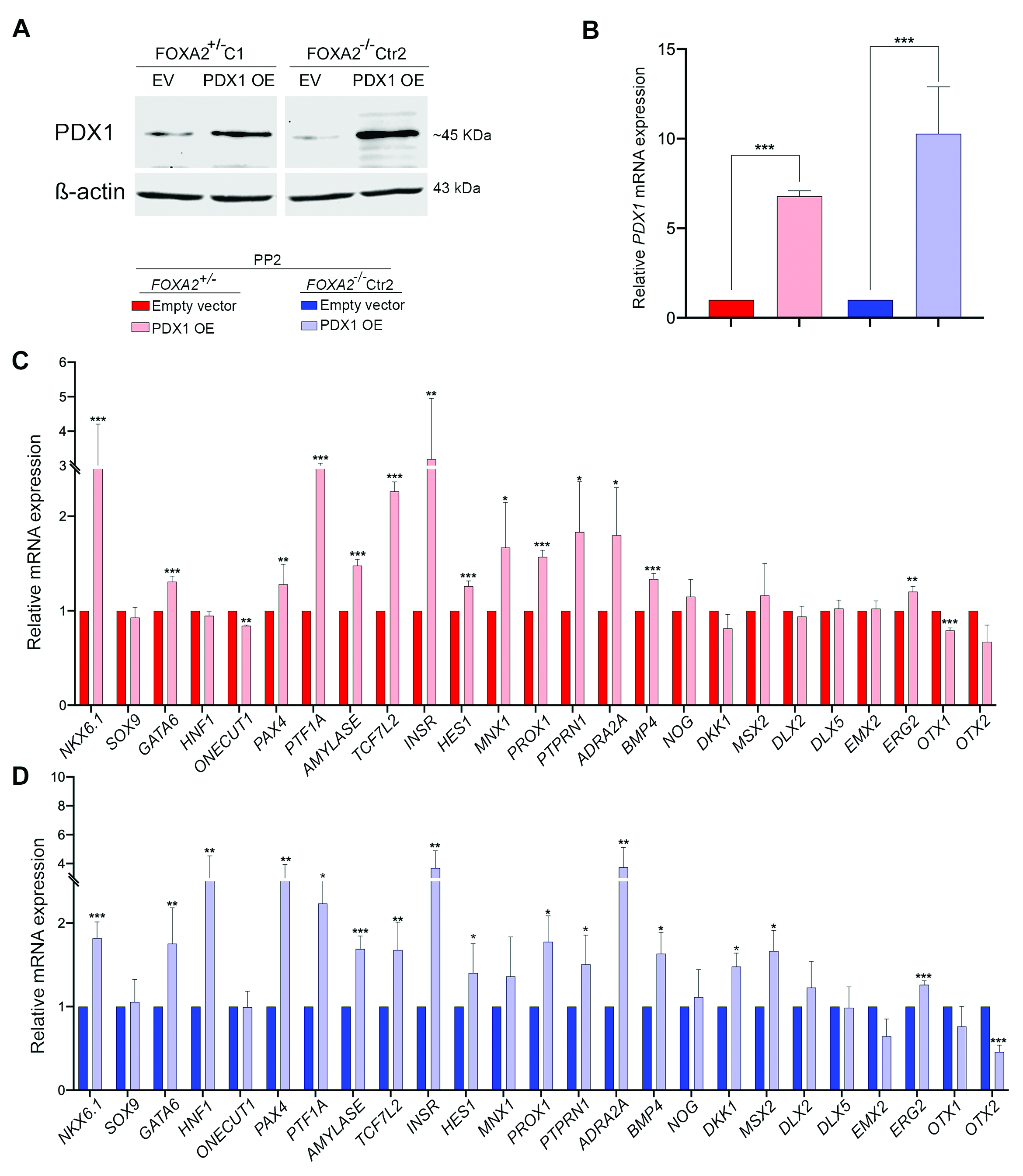

Supplement: Supplementary file 13 — Supplementary Figure 5 [file 41419_2021_3390_MOESM13_ESM.tif]

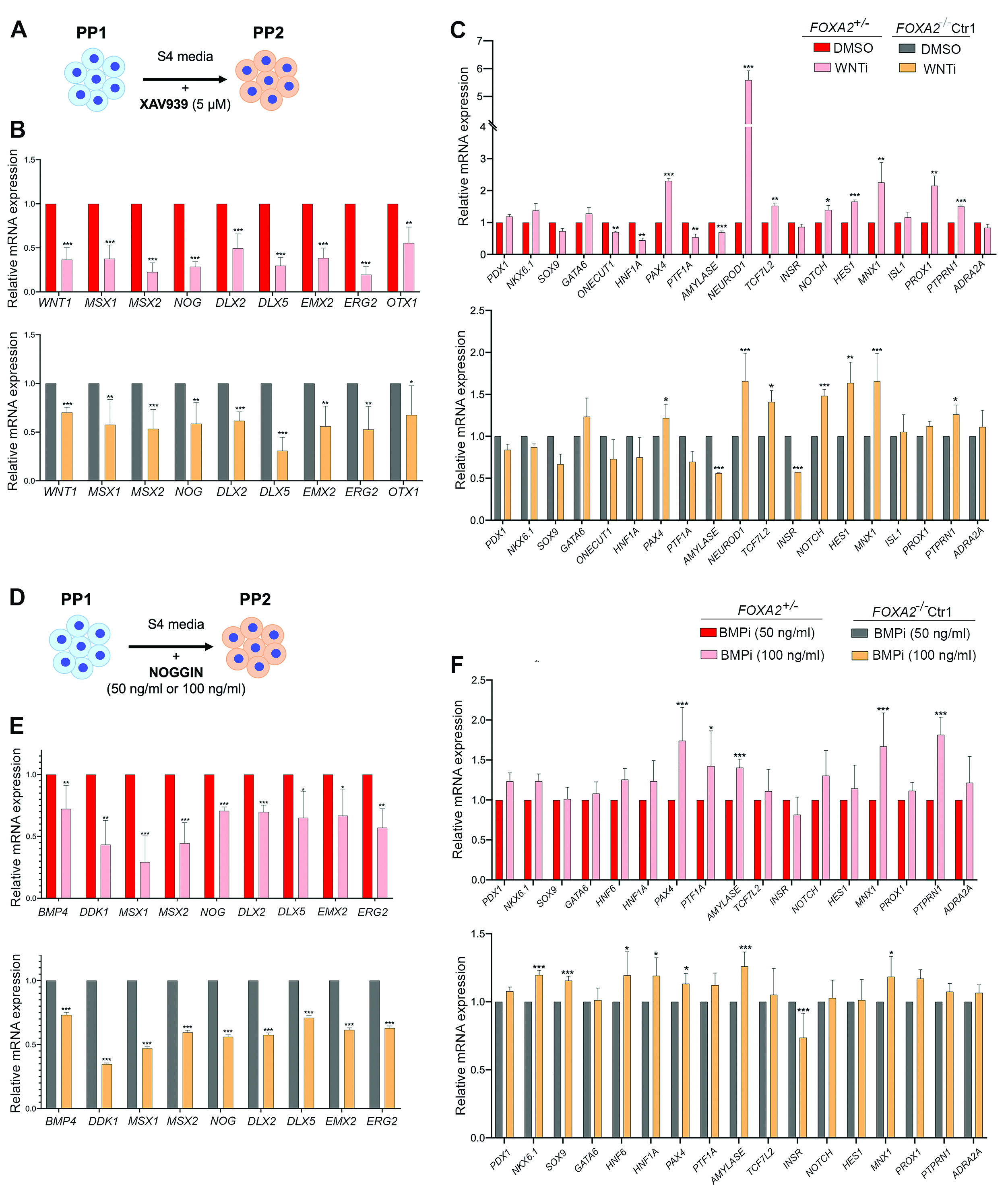

Supplement: Supplementary file 14 — Supplementary Figure 6 [file 41419_2021_3390_MOESM14_ESM.tif]
